# Tirbanibulin Attenuates Pulmonary Fibrosis by Modulating Src/STAT3 Signaling

**DOI:** 10.3389/fphar.2021.693906

**Published:** 2021-07-19

**Authors:** Xin Wang, Rui Ren, Zehui Xu, Haidi Huang, Wanglin Jiang, Jinbo Ma

**Affiliations:** ^1^ School of Pharmacy, Binzhou Medical University, Yantai, China; ^2^ Medicine & Pharmacy Research Center, Binzhou Medical University, Yantai, China

**Keywords:** pulmonary fibrosis, tirbanibulin, p-Src, HIF-1α, P-STAT3

## Abstract

Tirbanibulin (KX-01) is the first clinical Src inhibitor of the novel peptidomimetic class that targets the peptide substrate site of Src providing more specificity toward the Src kinase. This study assessed the impact of KX-01 on cobalt chloride (CoCl_2_)-treated L929 cells and bleomycin (BLM)-induced pulmonary fibrosis in rats to evaluate the efficacy of this compound *in vitro* and *in vivo*, respectively. In CoCl_2_-treated L929 cells, KX-01 significantly reduced the expression of smooth muscle actin (α-SMA), collagen I, collagen III, hypoxia inducing factor (HIF-1α), signal transducers and transcriptional activators (p-STAT3), and p-Src. In BLM-induced pulmonary fibrosis rats, KX-01 reduced pathological scores, collagen deposition, α-SMA, collagen I, collagen III, p-Src, HIF-1α, and p-STAT3. Overall, these findings revealed that KX-01 can alleviate experimental pulmonary fibrosis via suppressing the p-SRC/p-STAT3 signaling pathways.

## Introduction

Idiopathic pulmonary fibrosis (IPF) is a serious and progressive disorder that leads to progressive interstitial lung destruction (Attanoos et al., 2016). Rates of IPF have been rising in recent years, affecting approximately 3% of individuals over the age of 60 and causing high morbidity and mortality (Raghu et al., 2014). Indeed, IPF is associated with a five-year survival rate of just 20% and a mortality rate of approximately 50% owing to the lack of any specific treatment for this condition (Du et al., 2013; Chen et al., 2017).

IPF is characterized by fibroblast proliferation coupled with abnormal collagen and extracellular matrix deposition, tissue destruction, and inflammatory cell infiltration (Gao et al., 2012). Current therapeutic efforts aim to slow progressive pulmonary functional decline in individuals with less severe IPF (Somogyi et al., 2019). For example, nintedanib and pirfenidone can improve lung function despite adverse gastrointestinal side effects, but the data for reducing patient death are limited. To date, no drugs have been developed that can reliably reduce pulmonary fibrosis-associated mortality. As such, it is essential that the pathology of IPF be better clarified to guide the development of novel efficacious targeted compounds capable of treating this deadly disease.

Src is a non-receptor tyrosine kinase that is expressed within cells and that integrates myriad extracellular signaling inputs (Gerhard et al., 2019). Activation of Src can drive the proliferation and activation of fibroblasts by promoting the phosphorylation of STAT3, which, in turn, upregulates the expression of a range of genes associated with fibrotic phenotypes including HIF-1α. HIF-1α is, in turn, a key mediator of hypoxia-associated fibroblast proliferation in the context of pulmonary fibrosis (Brunton and Creedon, 2012; Lee et al., 2011; Prele et al., 2012). Tirbanibulin (KX-01) (Kempers et al., 2020) is a first-in-class peptidomimetic non-ATP kinase inhibitor capable of targeting the Src substrate-binding site, inhibiting Src kinase activity and downstream signaling activity (Fallah-Tafti et al., 2011; Anbalagan et al., 2012; Lamar et al., 2019). To date, no studies have investigated the therapeutic utility of KX-01 in models of experimental pulmonary fibrosis. Therefore, we aimed to explore the impact of KX-01 on an experimental model of pulmonary fibrosis both *in vitro* and *in vivo* and to determine the mechanistic basis for its activity.

## Materials and Methods

### Chemicals and Reagents

Tirbanibulin (KX-01, purity>98%, C_26_H_29_N_3_O_3_, CAS No: 897016-82-9) and the HIF-1α inhibitor Roxadustat (ROT) (purity>98%, C_19_H_16_N_2_O_5_, CAS No: 38808118-40-3) were from Shanghai Hanxiang Biomedical Company (China). The STAT3 inhibitor Stattic (purity>98%, C_8_H_5_NO_4_S, CAS No: 19983-44-9) was from the Jiangsu Aikang Biomedicine Company (China). Antibodies specific for p-STAT3, STAT3, HIF-1α, collagen I, collagen III, and α-SMA were obtained from the Shanghai Abcam Company (China). Secondary HRP-labeled goat anti-rabbit IgG (A0208), HRP-labeled goat anti-mouse IgG (AA128), mouse monoclonal anti-actin (A030-2-1), and C0088L were from Beyotime Biotechnology (China). A Masson’s trichrome staining kit (G1340) was purchased from the Beijing Solebao Technology Company (China). A hydroxyproline (Hyp) detection kit (A030-2-1) was obtained from the Nanjing Jiancheng Bioengineering Institute (Naniing, China). In addition, a TMB-based BeyoClick™ EdU cell proliferation assay kit was purchased from Beyotime Biotechnology.

### Establishment of a Rat Model of Bleomycin -Induced Pulmonary Fibrosis

In total, 24 healthy male Sprague Dawley rats (200–220 g) were housed in a climate-controlled animal facility (22 ± 2°C, 60 ± 10% relative humidity) with free food and water access. Following a 7 days acclimatization period, a model of BLM-induced pulmonary fibrosis was established in these rats (Huang et al., 2020). Briefly, rats were anesthetized and an endotracheal infusion of BLM (6 mg/kg in 0.2 ml normal saline) was administered. Sham control rats received an equivalent volume of normal saline. On day 22 (Histological examination showed obvious fibrosis in the lung) post-BLM administration, the 16 BLM-treated pulmonary fibrosis model rats were randomly separated based on body weight into the BLM model and KX-01 treatment groups. Animals in the KX-01 group received intragastric KX-01 (1.5 mg/kg/day for 14 days; dose determined to decrease the lung coefficient in the pre-experiment), while all other animals received an equivalent volume of sodium carboxymethyl cellulose. On day 36 (In general, 3 weeks after bleomycin induced pulmonary fibrosis in rats, then the corresponding drugs were given 2 weeks, pulmonary fibrosis could be significantly relieved), animals were euthanized and lung tissue samples were collected. Lung coefficient values were calculated as follows: lung coefficient = wet lung weight/body weight × 100%. After weighing, the lung tissue samples were separated into two portions, with the right lung snap-frozen for Western blotting analysis and the left lung fixed in 4% paraformaldehyde (PFA) prior to histological analysis.

### Histopathological Analysis

The center one-third of the left lung from each rat was fixed for 48 h in 4% PFA, dehydrated, paraffin-embedded, and cut into 4 μm-thick sections (Huang et al., 2020). The sections were then transferred onto poly-lysine-coated slides, deparaffinized using xylene, rehydrated with an ethanol gradient, and stained with hematoxylin and eosin (H&E). The degree of interstitial fibrosis in each section was then scored from 0 to 8 as follows: grade 0, normal lung; grade 1, isolated alveolar septa with subtle fibrotic changes; grade 2, fibrotic changes of alveolar septa with knot-like formation; grade 3, contiguous fibrotic walls of alveolar septa; grade 4, single fibrotic masses; grade 5, confluent fibrotic masses; grade 6, large contiguous fibrotic masses; grade 7, air bubbles; and grade 8, fibrous obliteration. Scoring was done separately in a blinded manner. Masson’s trichrome was used to measure collagen deposition, and the percentage of pulmonary fibrosis was assessed as previously described (Zhang et al., 2019).

### Immunohistochemical Staining

Pulmonary α-SMA and p-STAT3 expression was assessed via immunohistochemical staining. Briefly, 4 μm-thick lung tissue sections were deparaffinized, rehydrated, and treated for 10 min with 0.01 M citric acid in a 400 W steam microwave, after which 5% H_2_O_2_ in methanol was applied to quench endogenous peroxidase activity for 30 min at room temperature while protected from light. Sections were then blocked with 5% normal goat serum, after which they were probed overnight with rabbit polyclonal antibodies specific for α-SMA and p-STAT3 at 4°C, followed by probing for 30 min with HRP-conjugated anti-rabbit IgG at 37°C. Samples were then visualized via light microscopy. In total, five rats per group were analyzed, with three sections per rat being assessed. The Image-Pro Plus software (Media Cybernetics) was used to analyze tissue section images.

### Quantification of Hydroxyproline Levels

Lung tissue samples (30–100 mg) were added to test tubes and 1 ml of hydrolysate was added and thoroughly mixed. Samples were boiled for 20 min in a water bath, after which the Hyp contents in the samples were measured using the Hyp kit, according to the manufacturer’s instructions, with absorbance measured at 550 nm.

### Western Blotting

Snap-frozen lung tissue sections or cell samples were lyzed using radioimmunoprecipitation (RIPA) buffer containing protease inhibitors, after which supernatant protein concentrations were assessed using a BCA assay. Samples of protein (50 μg) were then separated via 8–10% SDS-PAGE, and the resultant blots were probed with antibodies specific for HIF-1α, p-STAT3, p-Src, α-SMA, collagen I, collagen III, and β-actin. ImageJ software was used to quantify protein band density, and β-actin density was used as a loading control to normalize assay results.

### Cell Culture

Murine L929 lung fibroblasts from the Chinese Academy of Sciences (Beijing, China) cell bank were cultured in MEM supplemented with 10% newborn calf serum and penicillin/streptomycin in a 37°C, 5% CO_2_, 95% N_2_ incubator. Cells were initially plated at 1×10^5^ /ml and were passaged every 3–4 days.

### Cell Proliferation Analysis

L929 cells were plated at 8×10^2^ cells/well in 96-well plates and incubated overnight at 37°C, after which the media were replaced with media containing a range of KX-01 concentrations (0, 0.01, 0.1 μM) supplemented with or without CoCl_2_ (100 nM), after which cells were incubated for 72 h. In other analyses exploring the mechanistic basis for observed pulmonary fibrosis-related phenotypes, cells were cultured with CoCl_2_ without or without 10 μM ROT (HIF-1α inhibitor) or 1 mM Stattic (STAT3 inhibitor) for 72 h. The BeyoClick™ 5-ethynyl-2′-deoxyuridine (EdU) Cell Proliferation Kit with TMB was used to measure cell proliferation. Absorbance values measured at 630 nm were normalized against those for untreated cells.

### L929 Cell Treatment for Western Blotting Analyses

L929 cells were grown until 60% confluent after which they were treated with KX-01 (0.01 μM) plus CoCl_2_ (100 nM) or were left untreated for 72 h. Alternatively, the mechanistic basis for pulmonary fibrosis was assessed by treating these cells with CoCl_2_ (100 nM) for 72 h with or without ROT (10 μM) or Stattic (1 μM). The expression of p-STAT3, p-Src, STAT3, HIF-1α, α-SMA, collagen I, and collagen III in these cells was then assessed via Western blotting.

### Statistical Analysis

Statistical analysis was performed using GraphPad Prism 5.0. Lung histopathology scores were compared between groups using the Wilcoxon rank-sum test. One-way ANOVAs with Dunnett’s test were used to compare all other data between groups. Data are given as means ± standard deviation. *p* < 0.05 was the significance threshold for this study.

## Results

### The Impact of KX-01 Treatment on Pulmonary Fibrosis-Associated Lung Pathology

**FIGURE 1:**
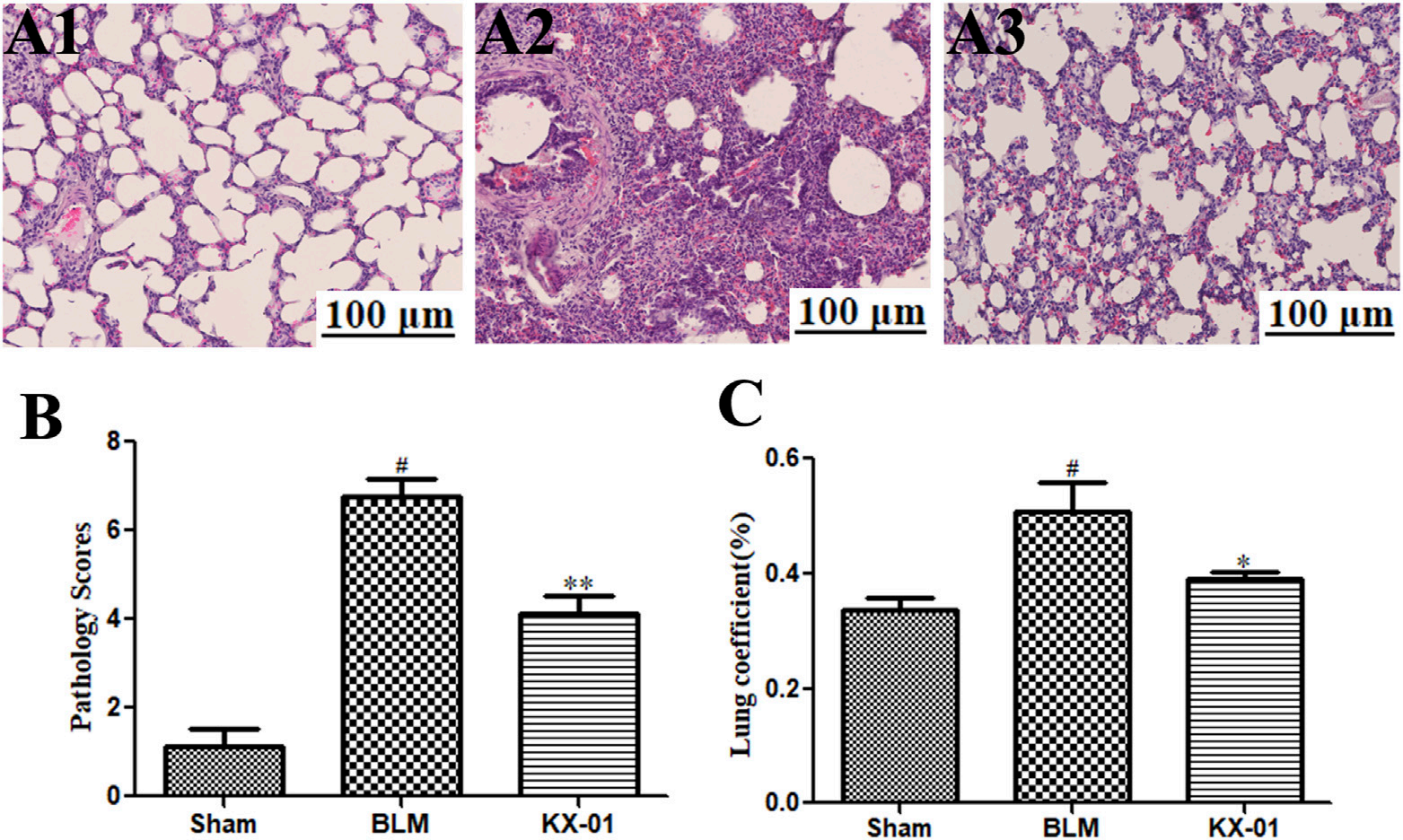
The impact of KX-01 on lung tissue histopathology. **(A)** Representative hematoxylin and eosin (H&E)-stained tissue sections **(A1–A3)**. **(B)**: The impact of KX-01 on lung histopathology scores. **(C)**: The impact of KX-01 on BLM-induced changes in lung coefficient values in this rat pulmonary fibrosis model system. BLM: bleomycin. Data are means ± SD (*n* = 8). ^#^ *p* < 0.01 vs. sham; ^*^ *p* < 0.05, ^**^ *p* < 0.01 vs. BLM. Data were compared via ANOVAs with Dunnett's test.

We began by evaluating fibrosis in our rat BLM-induced pulmonary fibrosis model system. As expected, sham control rats exhibited minimal alveolar inflammatory cell infiltration (Figure 1A), whereas rats in the BLM model group exhibited significant inflammatory cell infiltration, fibrosis, and alveolar tissue destruction (Figure 1A2). Rats that had been treated with KX-01 showed reduced levels of inflammatory cell infiltration and pulmonary interstitial thickening, with significantly lower pathological scores relative to the model group animals (Figures 1A3,B). We additionally measured lung coefficient values to quantify the degree of pulmonary fibrosis in these rats. During the early stages of pulmonary fibrosis, this ratio rises due to an increase in pulmonary mass attributable to cellular swelling and capillary congestion, whereas during later stages it is primarily due to collagen fiber accumulation. Relative to BLM-treated model group rats, lung coefficient values in KX-01-treated rats were significantly decreased (Figure 1C).

### The Impact of KX-01 on Pulmonary Collagen Deposition

**FIGURE 2:**
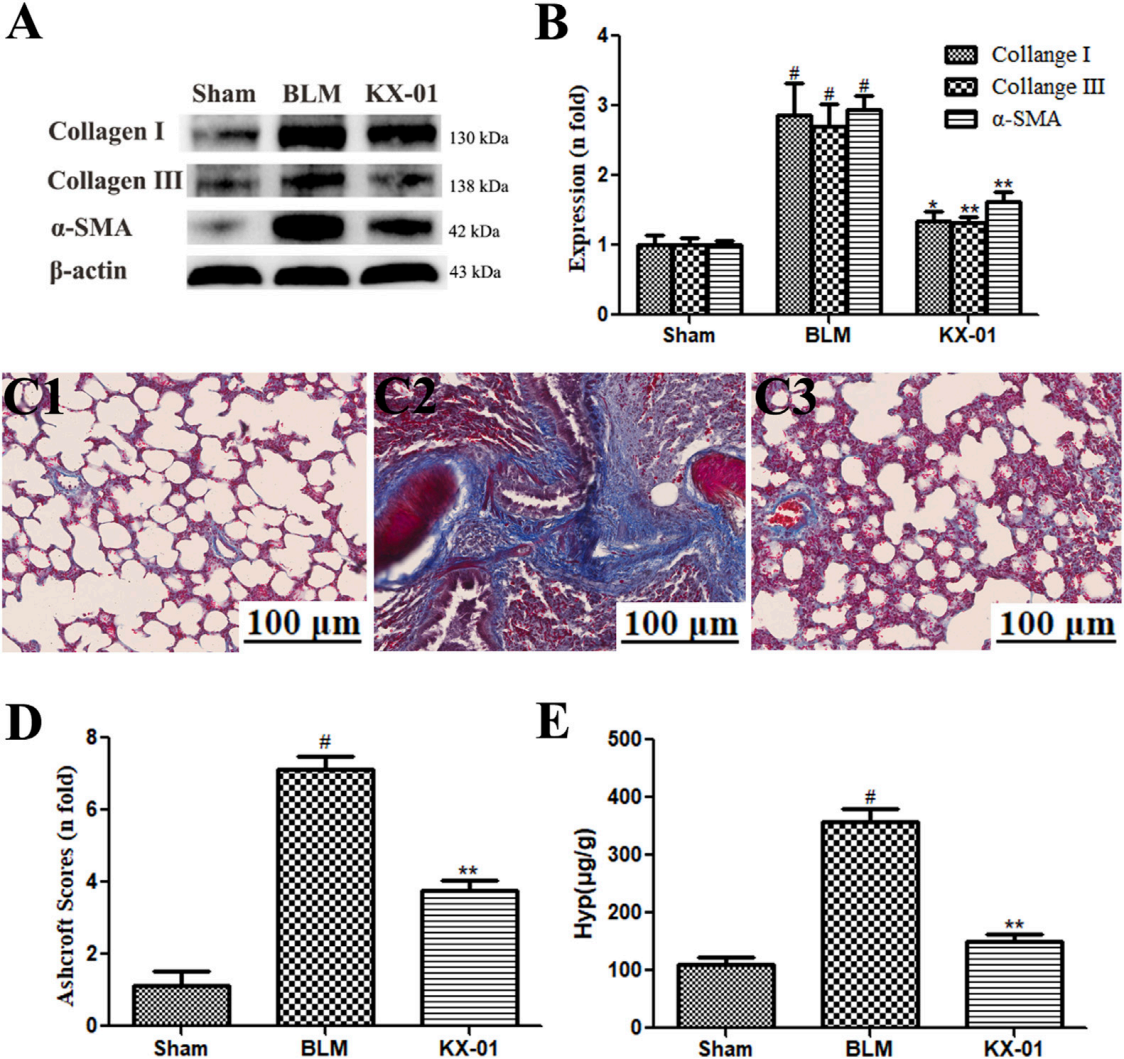
The impact of KX-01 on pulmonary collagen deposition. **(A, B)**: The impact of KX-01 on collagen content and α-SMA expression; **(C, D)**: Masson’s trichrome staining **(C1–C3, D)**; **(E)**: Hyp level in lung tissue. BLM: bleomycin; Hyp: hydroxyproline. Data are means ± SD (*n* = 8). ^#^ *p* < 0.01 vs. sham; ^*^ *p* < 0.05, ^**^ *p* < 0.01 vs. BLM. Data were compared via ANOVAs with Dunnett’s test.

Next, we assessed pulmonary collagen deposition in our experimental model system via Western blotting-mediated measurement of α-SMA, collagen I, and collagen III levels (Figures 2A,B) and Masson’s trichrome staining (Figures 2C,D). We detected substantial interstitial collagen deposition in BLM model rats with Masson’s trichrome staining, whereas collagen levels were significantly reduced in lung samples from KX-01-treated rats (Figure 2D). Hyp accounts for ∼13% of collagen amino acid content and can thus be measured as a means of quantifying collagen levels within a given tissue. Consistent with our staining results, we found that Hyp levels were significantly reduced in KX-01-treated rats relative to the BLM-treated model rats (Figure 2E). Western blotting further confirmed that pulmonary α-SMA, collagen I, and collagen III expression levels were significantly elevated in BLM-treated model rats relative to sham controls, while they were significantly reduced in KX-01-treated rats relative to BLM-treated model rats (Figures 2A,B).

The impact of KX-01 on the pulmonary expression of fibrosis-related proteins.

**FIGURE 3:**
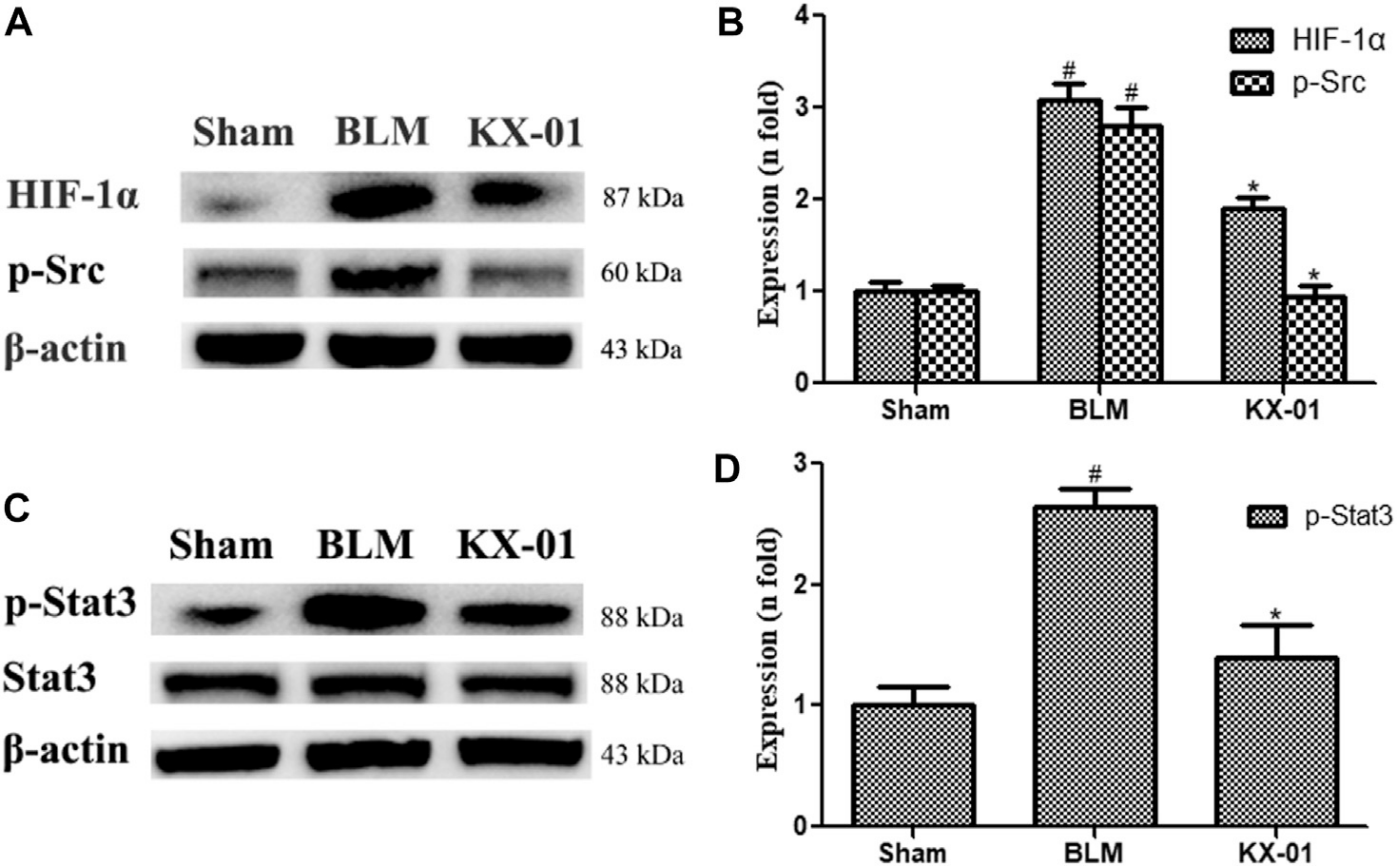
The impact of KX-01 on pulmonary protein expression. **(A, B)**: The expression of HIF-1α, p-Src; **(C, D)**: p-STAT3, STAT3 and p-STAT3/STAT3 was analyzed. BLM: bleomycin. Data are means ± SD (*n* = 3). ^#^ *p* < 0.01 vs. sham; ^*^ *p* < 0.01 vs. BLM. Data were compared via ANOVAs with Dunnett’s test.

We next analyzed pulmonary p-Src, p-STAT3, STAT3, and HIF-1α expression profiles by Western blotting. Relative to sham control rats, those in the BLM model group exhibited significant increases in HIF-1α, p-Src, and p-STAT3 protein levels, whereas all of these proteins were downregulated in KX-01-treated rats (Figures 3A–D). Additionally, overall pulmonary STAT3 expression levels were unaffected in response to BLM or KX-01 treatment (Figure 3C).

**FIGURE 4:**
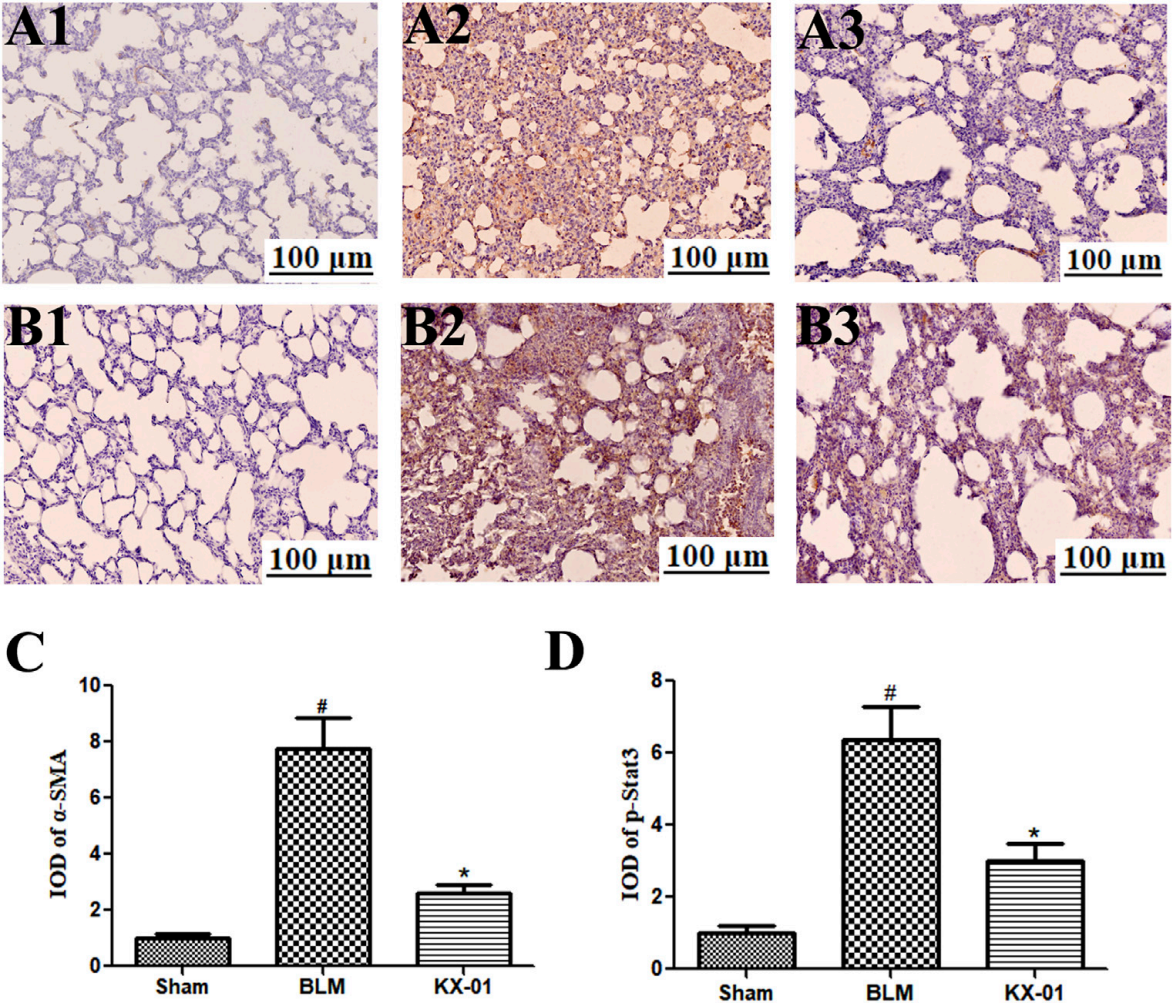
The impact of KX-01 on pulmonary α-SMA and p-STAT3 immunohistochemical staining. **(A)** and **(4C)**: Immunohistochemical staining was used to assess pulmonary α-SMA; **(B–D)**: Immunohistochemical staining was used to assess pulmonary p-STAT3 expression following BLM treatment with or without KX-01 administration. BLM: bleomycin. Data are means ± SD. ^#^ *p* < 0.01 vs. sham; ^*^ *p* < 0.01 vs. BLM. Data were compared via ANOVAs with Dunnett’s test.

The expression of the fibroblast markers α-SMA and p-STAT3 were assessed by immunohistochemical staining in the lungs of our model rats (Figures 4A,B). These analyses revealed that the expression of α-SMA and p-STAT3 was significantly increased in BLM-treated model rats relative to the sham controls whereas KX-01 treatment reversed these increases (Figures 4C,D).

### The Impact of KX-01 on L929 Cell Proliferation and Protein Expression

**FIGURE 5:**
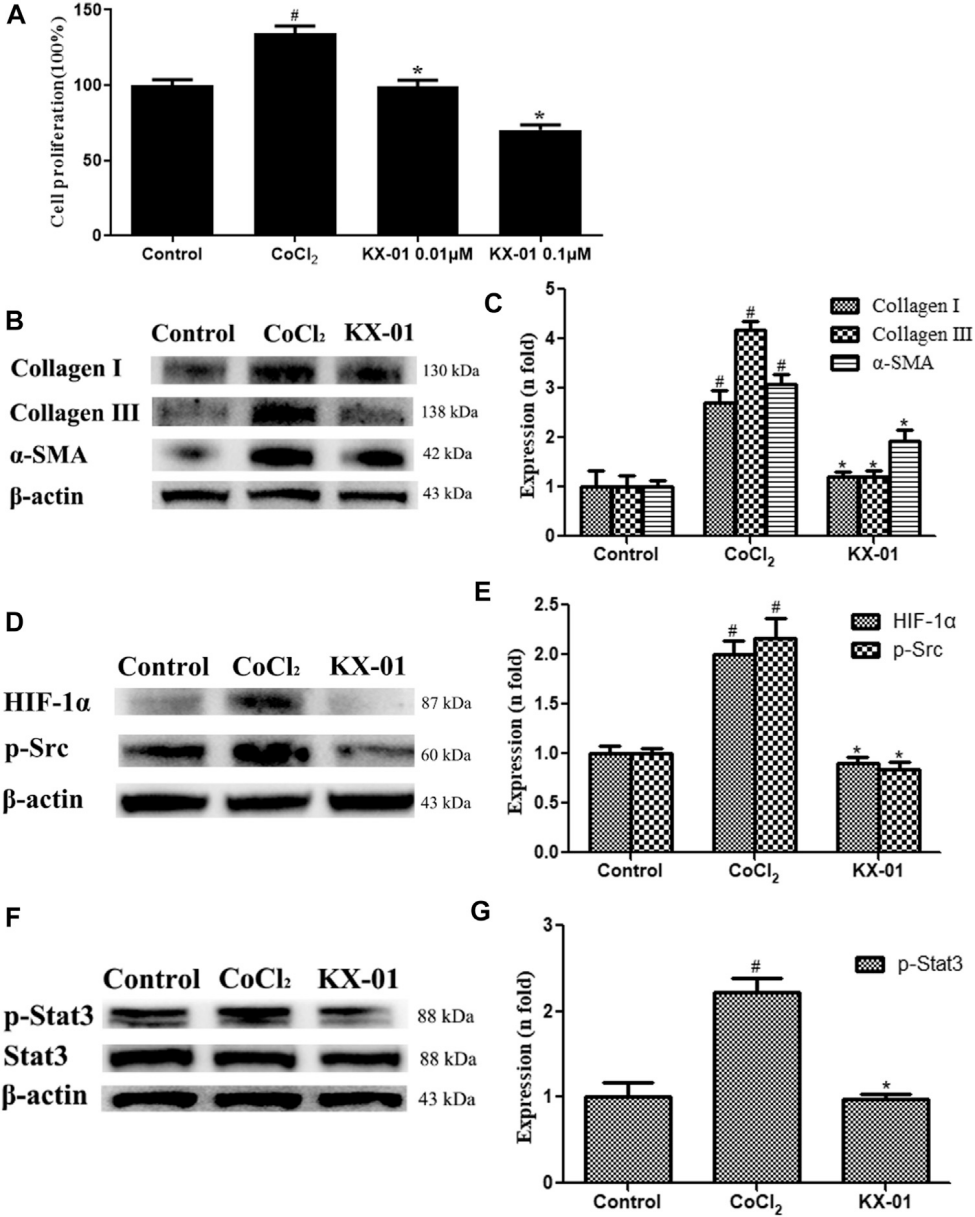
The impact of KX-01 on L929 cell proliferation and protein expression. **(A)** An EdU incorporation assay was used to assess L929 cell proliferation following CoCl_2_ treatment with or without KX-01 for 72 h. **(B–C)**: The expression of α-SMA, collagen I, and collagen III were assessed; **(D, E)**: The expression of HIF-1α, p-Src (D–E); **(F, G)**: The expression of p-STAT3 and STAT3, and the ratio of p-STAT3/STAT3 was assessed. Data are means ± SD (*n* = 3). ^#^ *p* < 0.01 vs. control; ^*^ *p* < 0.01 vs. CoCl_2_. Data were compared via ANOVAs with Dunnett's tst.

To expand upon *in vivo* findings, we established an *in vitro* model of pulmonary fibrosis by stimulating murine L929 cells with CoCl_2_ (100 nM) under anoxic conditions to mimic a fibrotic environment. At 72 h post-CoCl_2_ treatment, we then assessed the proliferation of these cells via an EdU incorporation assay, revealing that CoCl_2_ stimulation markedly enhanced cellular proliferation, whereas KX-01 (0.01 or 0.1 μM) markedly inhibited CoCl_2_-induced proliferation (Figure 5A). Therefore, subsequent *in vitro* assays on related proteins were studied using the 0.01 μM concentration. We found that collagen I, collagen III, and α-SMA protein levels were significantly increased in CoCl_2_-treated cells, whereas KX-01 suppressed this effect (Figures 5B,C). Similarly, CoCl_2_ promoted the upregulation of p-Src, p-STAT3, and HIF-1α relative to control cells while this was largely prevented by KX-01 treatment (Figures 5D–F) even to normal levels. In addition, no differences in the total STAT3 protein levels were observed in L929 cells as a function of CoCl_2_ or KX-01 treatment (Figures 5F,G).

**FIGURE 6:**
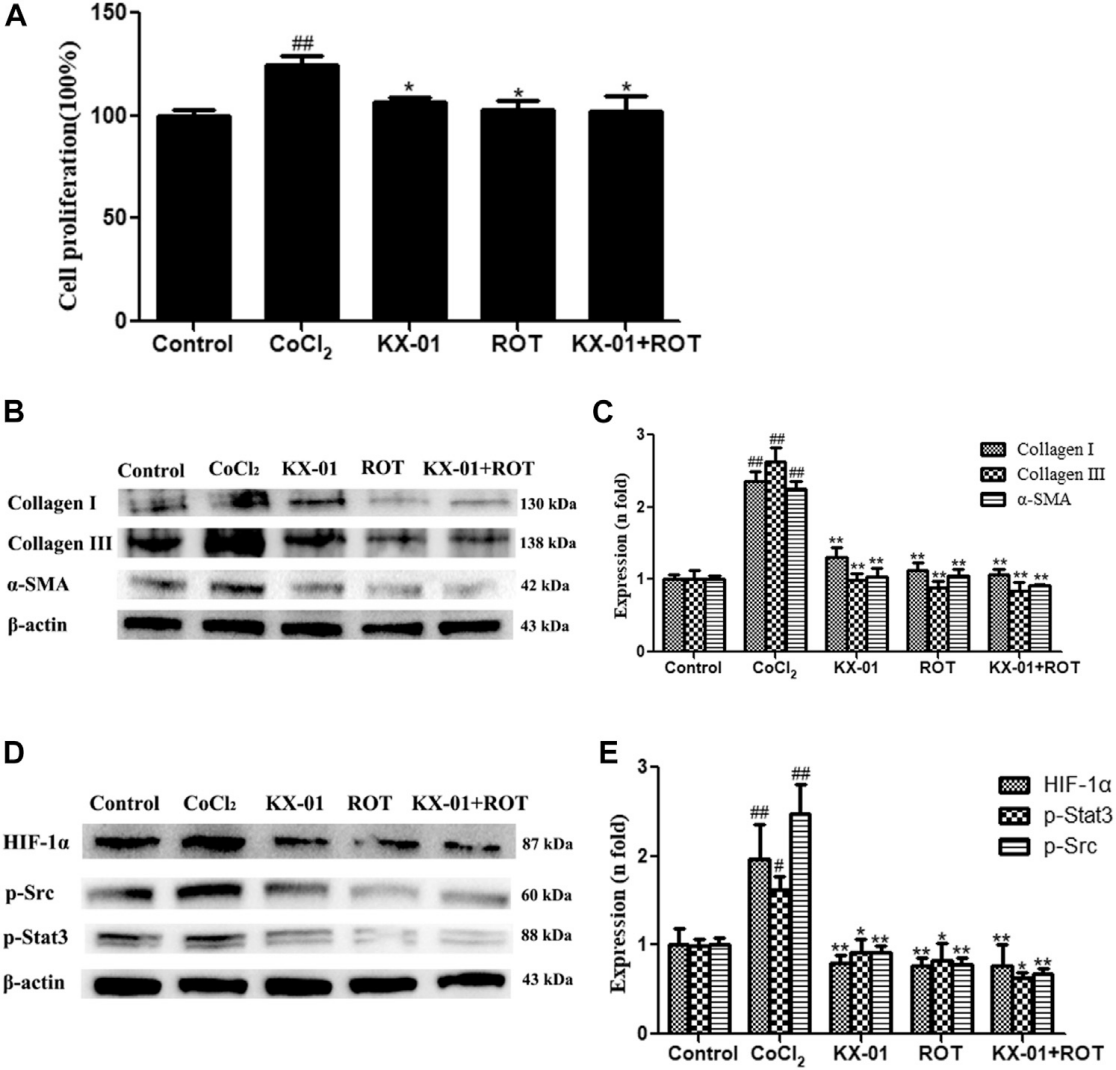
The impact of KX-01 on L929 cell proliferation and protein expression in the presence of Roxadustat. **(A)** An EdU incorporation assay was used to assess L929 cell proliferation following CoCl_2_ treatment with or without KX-01 or ROT for 72 h. **(B, C)**: The expression of α-SMA, collagen I, and collagen III were assessed; **(D, E)**: The expression of HIF-1α, p-Src, p-STAT3 and STAT3, and the ratio of p-STAT3/STAT3 were assessed. ROT: Roxadustat. Data are means ± SD (*n* = 3). ^#^ *p* < 0.05, ^##^ *p* < 0.01 vs. control; ^*^ *p* < 0.05, ^**^ *p* < 0.01 vs. CoCl_2_. Data were compared via ANOVAs with Dunnett's test.

To better understand the mechanisms whereby KX-01 mediates its anti-fibrotic activity, we next treated CoCl_2_-stimulated L929 cells with the HIF-1α inhibitor ROT (10 μM) in the presence or absence of KX-01 and then analyzed cellular proliferation (Figure 6A), collagen I, collagen III, α-SMA, p-STAT3, p-Src, and HIF-1α protein levels (Figures 6B–E). Relative to CoCl_2_-treated cells, KX-01 or ROT single-agent treatment suppressed L929 cell proliferation and decreased HIF-1α, p-Src, and p-STAT3 protein expression. No further reductions in proliferation or protein expression were observed in cells treated with both KX-01 and ROT, thus suggesting that KX-01 ameliorates experimental pulmonary fibrosis at least in part by inhibiting the activity of HIF-1α (Figures 6D,E and Figure 8).

**FIGURE 7:**
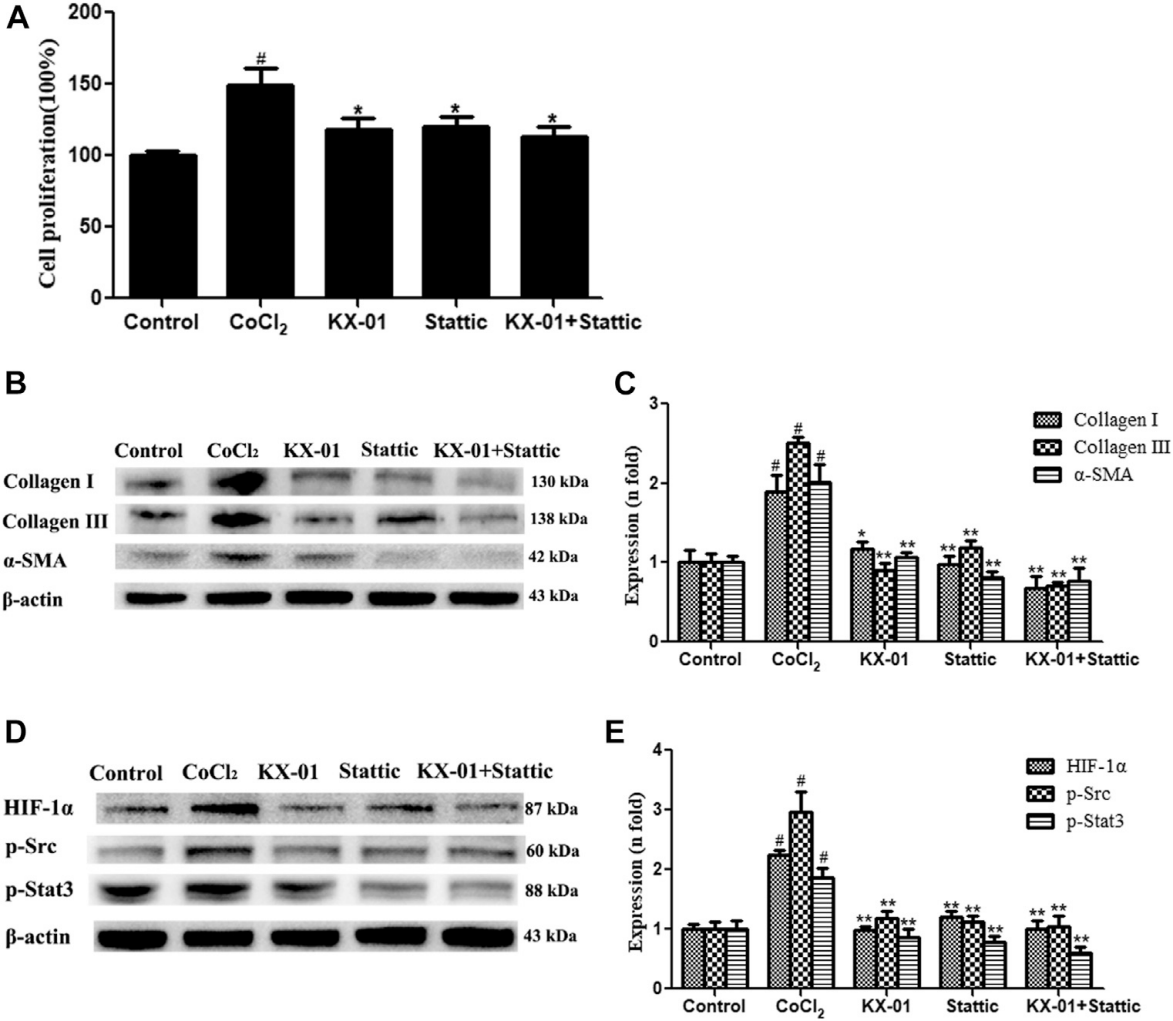
The impact of KX-01 on L929 cell proliferation and protein expression in the presence of Stattic. **(A)** An EdU incorporation assay was used to assess L929 cell proliferation following CoCl_2_ treatment with or without KX-01 or Stattic for 72 h. **(B, C)**: The expression of α-SMA, collagen I, and collagen III were assessed; **(D, E)**: The expression of HIF-1α, p-Src, p-STAT3 and STAT3, and the ratio of p-STAT3/STAT3 were assessed. Data are means ± SD (*n* = 3). ^#^ *p* < 0.01 vs. control; ^*^ *p* < 0.05, ^**^ *p* < 0.01 vs. CoCl_2_. Data were compared via ANOVAs with Dunnett's test.

**FIGURE 8:**
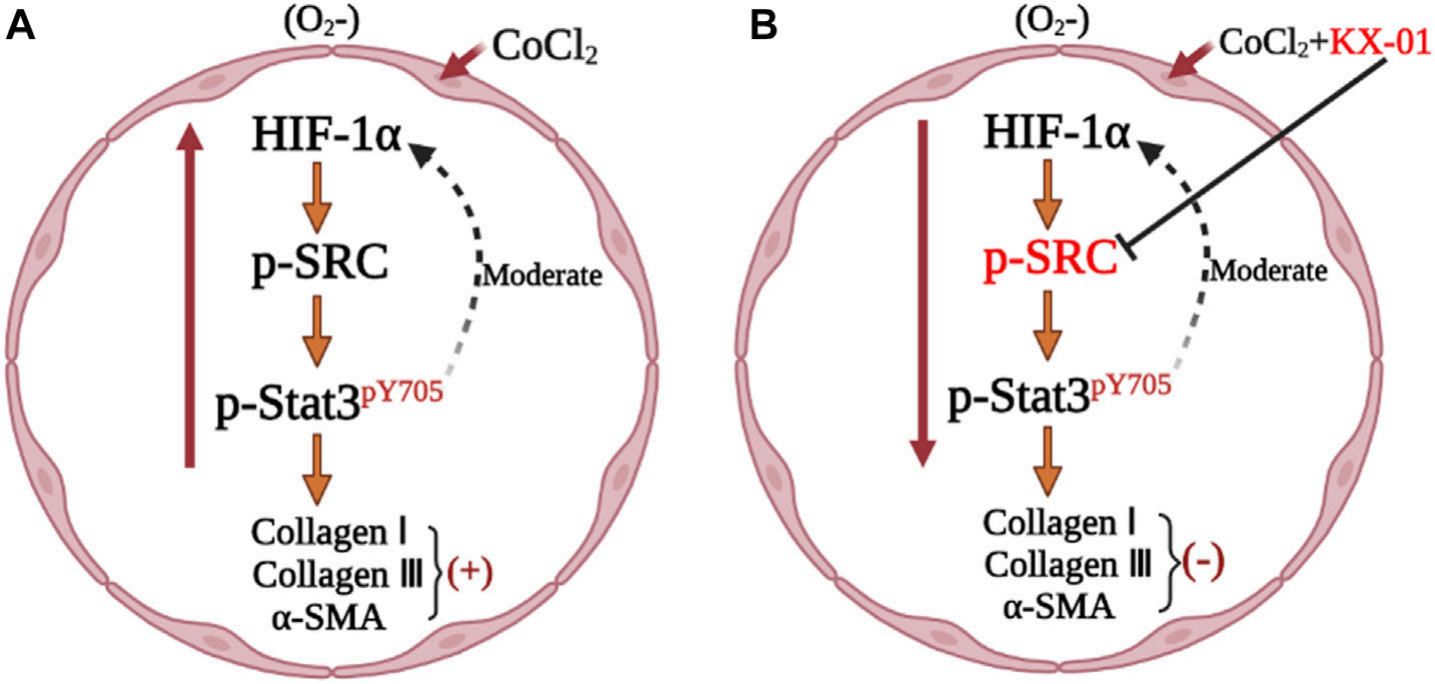
Mechanism of KX-01 on experimental pulmonary fibrosis.

We next evaluated the role of STAT3 activation in the context of KX-01 anti-fibrotic activity by treating CoCl_2_-stimulated L929 cells with the STAT3 inhibitor Stattic (1 μM) in the presence or absence of KX-01, after which we evaluated the proliferation (Figure 7A) and collagen I, collagen III, α-SMA, p-STAT3, p-Src, and HIF-1α protein expression in these cells (Figures 7B–E). Relative to CoCl_2_-treated cells, KX-01 or Stattic single-agent treatment suppressed L929 cell proliferation and decreased HIF-1α, p-Src, and p-STAT3 protein expression. No further reductions in proliferation or protein expression were observed in cells treated with both Stattic and KX-01 plus Stattic, thus suggesting that KX-01 ameliorates experimental pulmonary fibrosis at least in part by inhibiting STAT3 activation (Figures 7D,E and Figure 8).

## Discussion

Idiopathic pulmonary fibrosis (IPF) is a progressive pulmonary disease associated with serious morbidity and poor prognosis. The etiological basis of IPF is complex, and it remains challenging to treat effectively (Saito et al., 2019), with most current therapeutic efforts being symptomatic or centered around slowing the overall disease progression. The identification of novel therapeutic targets and strategies capable of preventing or alleviating this disease is thus essential.

α-SMA is a specific marker of myofibroblasts, which are derived from fibroblasts and are associated with poorer IPF patient survival (Waisberg et al., 2012). Analyses of extracellular matrix (ECM) samples from IPF patients demonstrate that interstitial type I and type III collagen levels increase during the early stages of pulmonary fibrosis, with type III collagen being found within the interstitium and thickened alveolar septum, while type I collagen, which accounts for ∼80% of the total collagen content in IPF, is found in regions of tissue fibrosis (Glasser et al., 2016). Hyp levels can be measured to quantitatively gauge the extent of collagen deposition (Tanaka et al., 2010), which in turn reflects the degree of IPF severity and the efficacy of anti-fibrotic drugs. KX-01 is a first-in-class peptidomimetic Src inhibitor that specifically targets the Src substrate-binding site (Anbalagan et al., 2012). This study is the first to our knowledge to have assessed the anti-fibrotic activity of KX-01 in a preclinical model of pulmonary fibrosis as a means of exploring its potential utility for the clinical treatment of IPF. We ultimately found that KX-01 was able to markedly alleviate pulmonary fibrosis by decreasing fibrosis-related increases in α-SMA, Hyp collagen I, and collagen III levels within the lungs.

IPF is a proliferative disorder driven by chronic hypoxia that becomes progressively more severe during the later stages of this disease. Hypoxia-mimicking conditions were established by CoCl_2_, characterized by the nuclear accumulation of HIF-1a. As such, we utilized an *in vitro* model of IPF wherein cells were cultured in the presence of CoCl_2_ under hypoxic conditions rather than TGF-β1 (Huang et al., 2020), as this has been shown to induce fibroblast proliferation and nuclear HIF-1α accumulation (Song et al., 2018). Src can regulate diverse signaling pathways within tumor cells, controlling survival, proliferation, angiogenesis, invasion, and related activities in oncogenic contexts (Brunton and Creedon, 2012).

Src also plays a role in the pathogenesis of IPF by influencing myofibroblast activation, inflammation, and the epithelial-mesenchymal transition, all of which are hallmarks of this fibrotic disease (Li et al., 2020). Src activates HIF-1α (Lee et al., 2011), and as such, KX-01 can disrupt HIF-1α accumulation and thereby alleviate pulmonary fibrosis. Consistent with the observed anti-fibrotic activity of KX-01, the dual-specificity Src/Abl family kinase inhibitor saracatinib has been granted orphan drug designation by the FDA for the treatment of IPF.

STAT3 is a transcription factor that exists in an inactive state in the cytoplasm under homeostatic conditions (Guanizo et al., 2018), but that plays a central role in governing the activation of pulmonary fibroblasts/myofibroblasts and the progression of IPF (Prele et al., 2012). When phosphorylated, STAT3 dimerizes and translocates to the nucleus wherein it is able to bind to specific gene promoter sequences to promote target gene transcription. As such, the p-STAT3/STAT3 ratio is a key indicator of fibroblast activation (Chakraborty et al., 2017). We found that KX-01 treatment was sufficient to reduce the p-STAT3/STAT3 ratio without affecting total STAT3 protein levels. Src family tyrosine kinases can facilitate basal STAT3 activation such that inhibiting Src kinase activity can suppress STAT3-DNA binding (Garcia et al., 2001). To date, KX-01 has successfully completed standard preclinical and phase I clinical trials (Antonarakis et al., 2013; Naing et al., 2013), and exhibits satisfactory pharmacokinetic properties when orally administered. In the present study, we found that KX-01 treatment was sufficient to reduce p-SRC and p-STAT3 levels and to thereby alleviate pulmonary fibrosis. However, there are still some limitations in this study. KX01 is an inhibitor of Src. Src is associated with inflammation, and inflammation is also associated with fibrosis, which needs to be further revealed in subsequent studies.

## Conclusion

In conclusion, we determined that KX-01 was able to suppress cellular proliferation, decrease the expression of HIF-1α, p-Src, α-SMA, collagen I, and collagen III, and lower the p-STAT3/STAT3 ratio *in vitro* and *in vivo,* suggesting that this compound may alleviate experimental pulmonary fibrosis by modulating the p-SRC/p-STAT3 signaling pathways.

## Data Availability

The original contributions presented in the study are included in the article/Supplementary Material, further inquiries can be directed to the corresponding authors.
