# Supplementary figures and images for: Tirbanibulin Attenuates Pulmonary Fibrosis by Modulating Src/STAT3 Signaling
Source: Front Pharmacol. 2021 Jul 19;12:693906. doi: 10.3389/fphar.2021.693906 (PMC8326405; doi:10.3389/fphar.2021.693906)

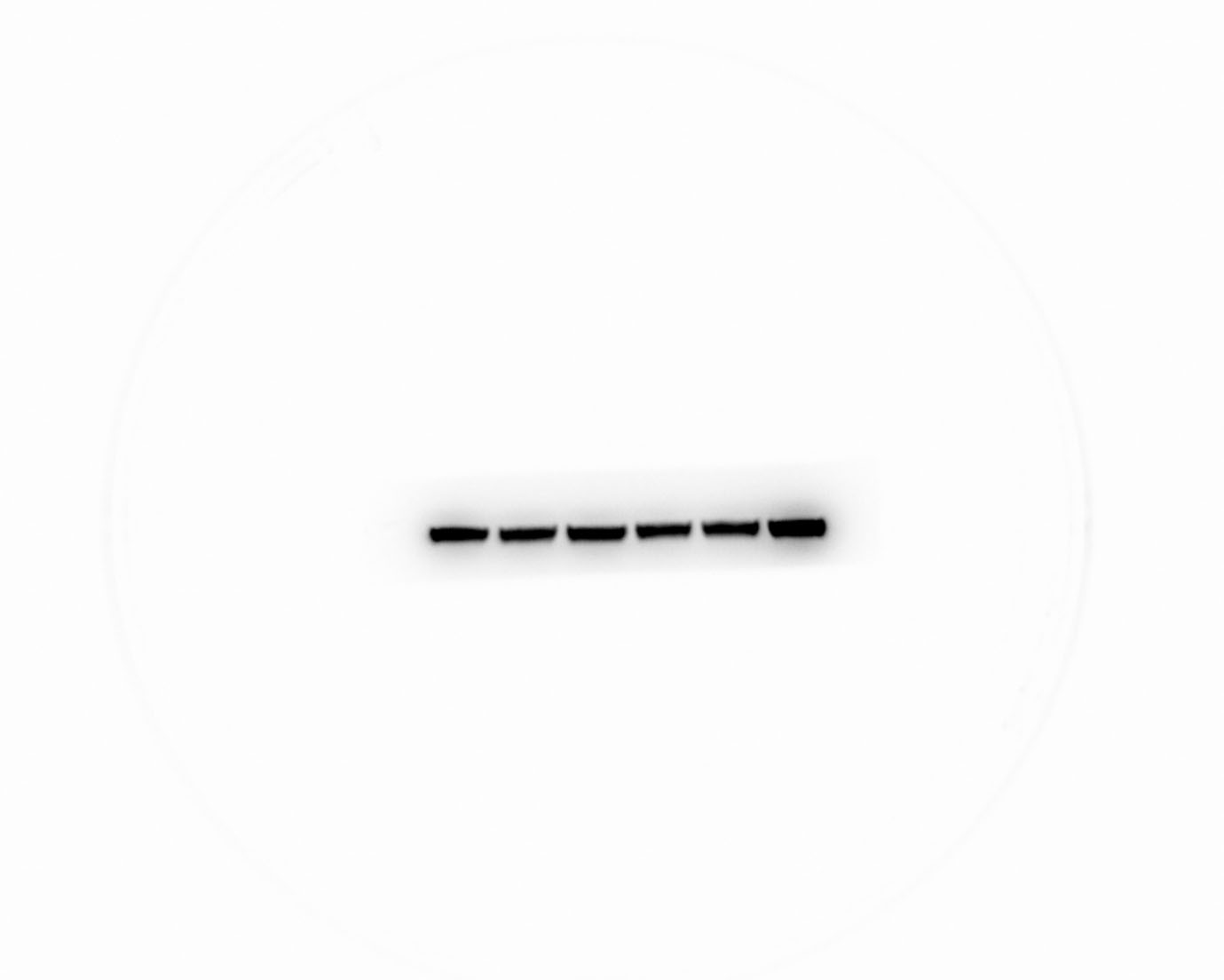

Supplement: Supplementary file 1 [file Image15.JPEG]

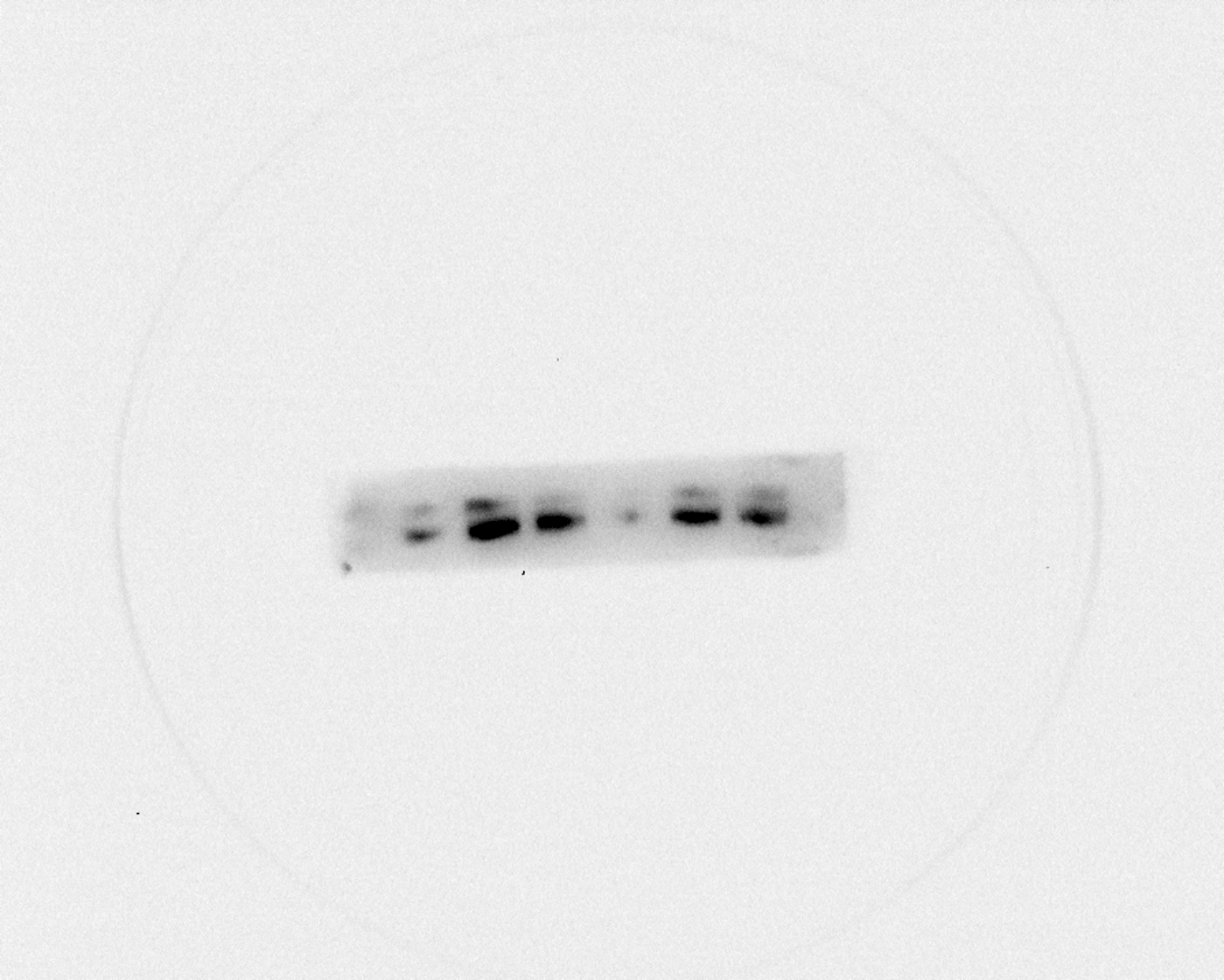

Supplement: Supplementary file 2 [file Image6.TIF]

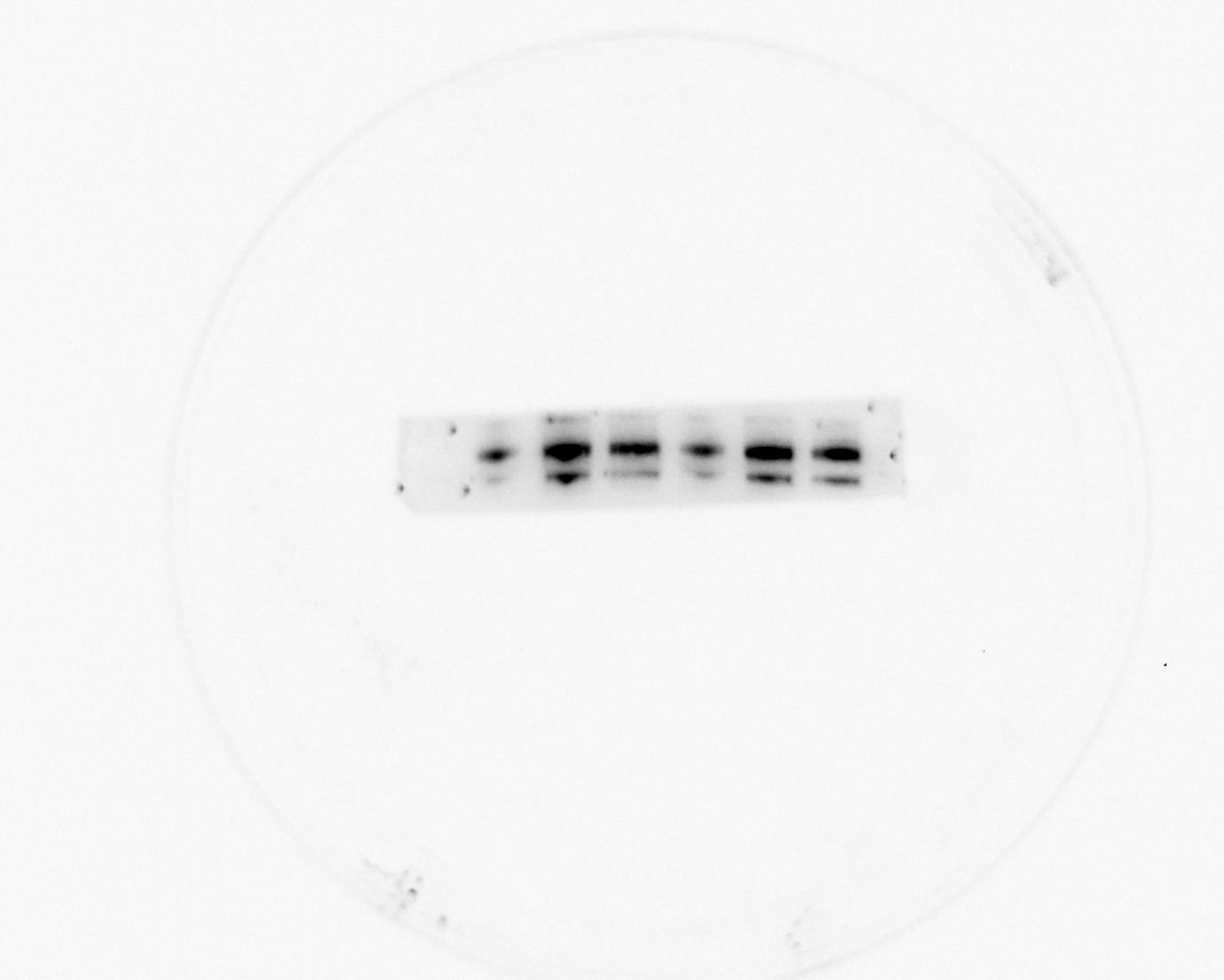

Supplement: Supplementary file 3 [file Image14.TIF]

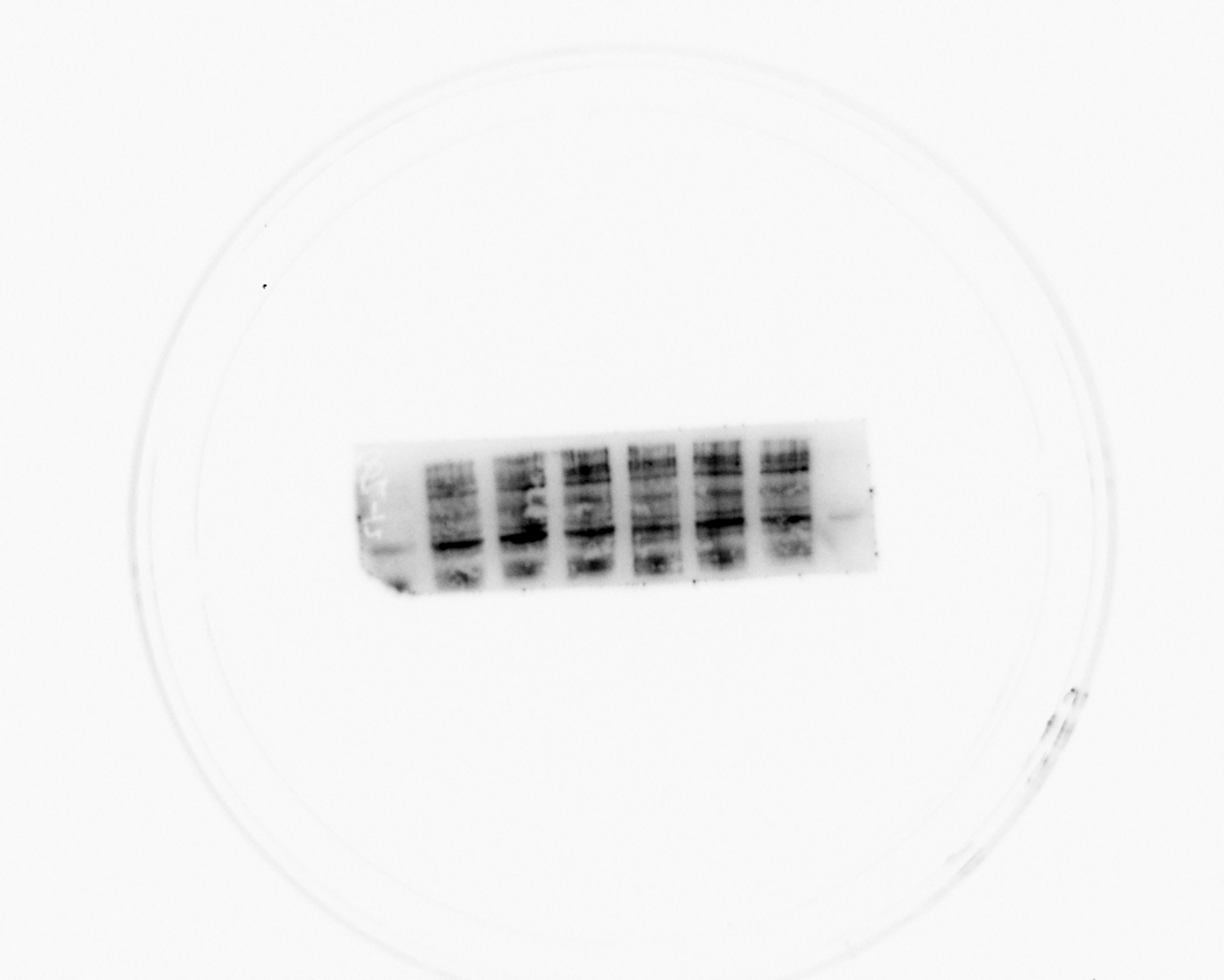

Supplement: Supplementary file 4 [file Image3.TIF]

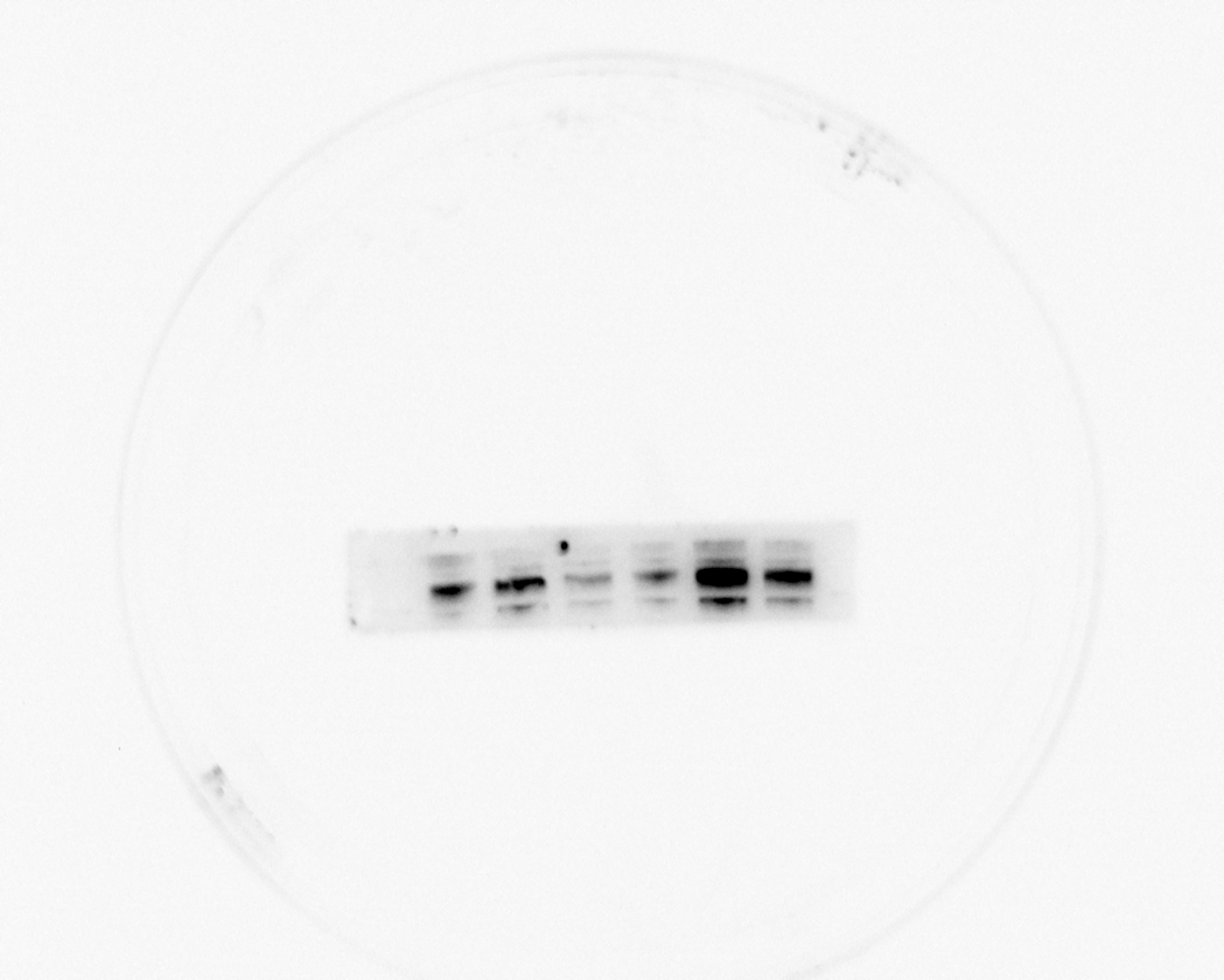

Supplement: Supplementary file 5 [file Image4.TIF]

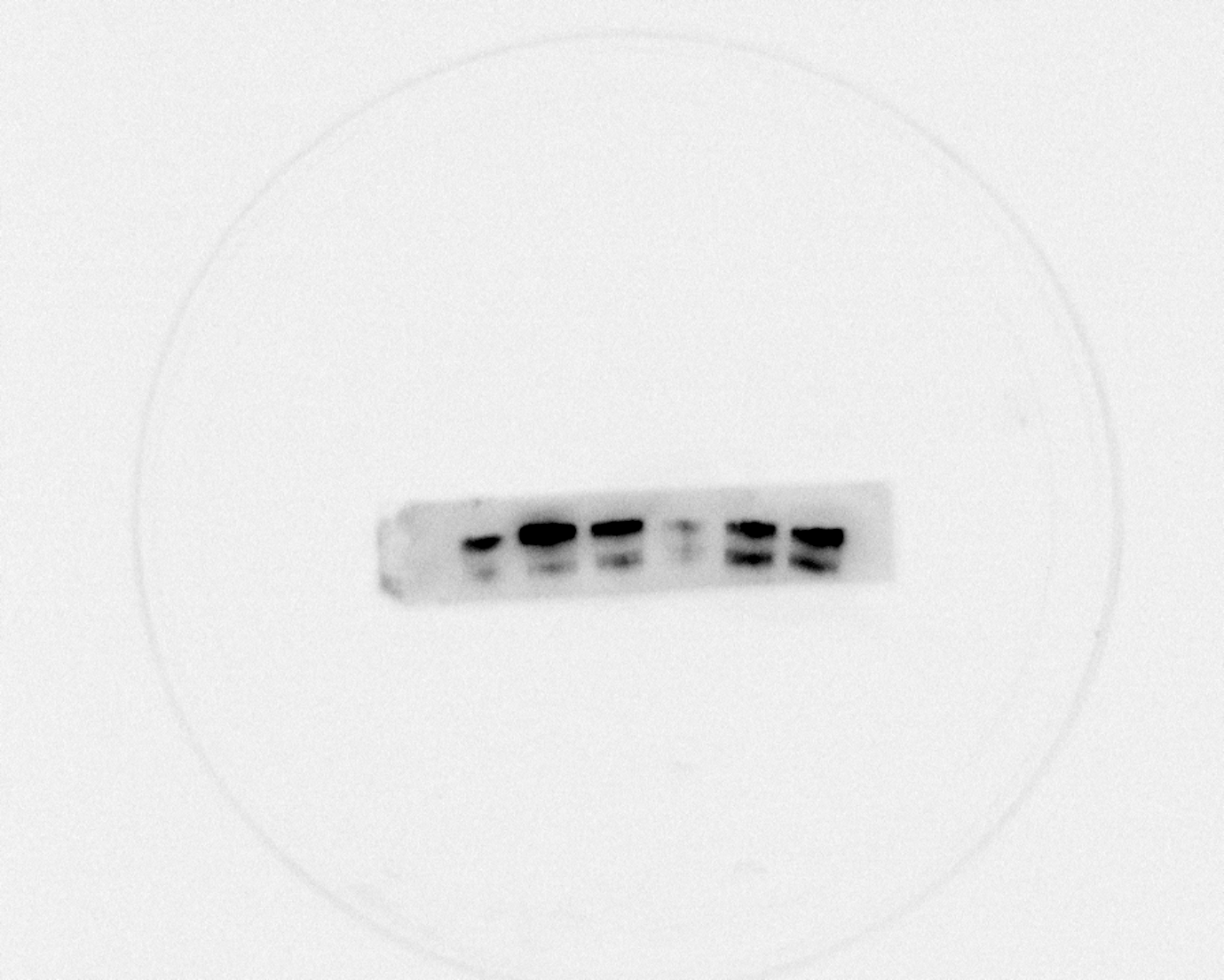

Supplement: Supplementary file 6 [file Image9.TIF]

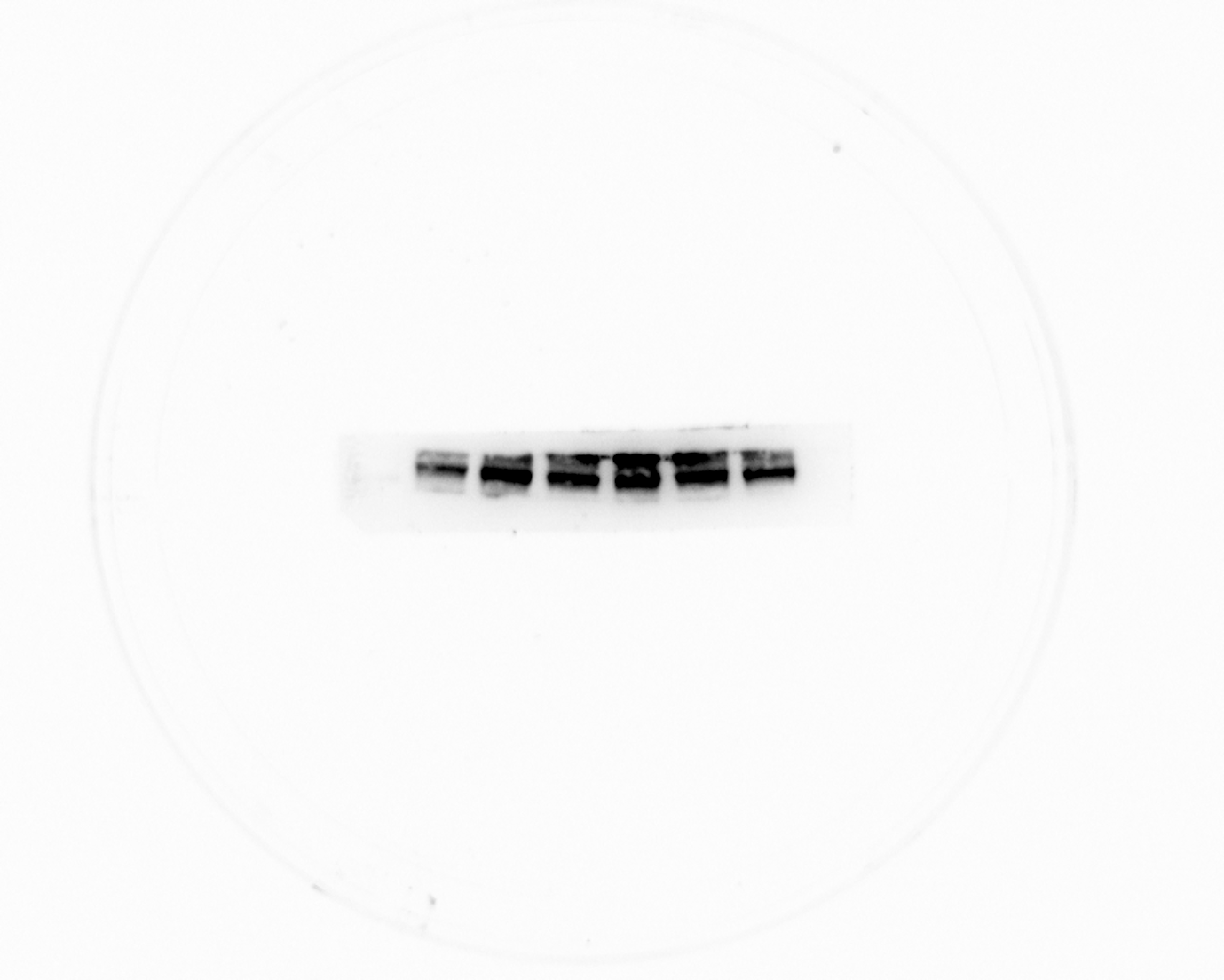

Supplement: Supplementary file 7 [file Image2.TIF]

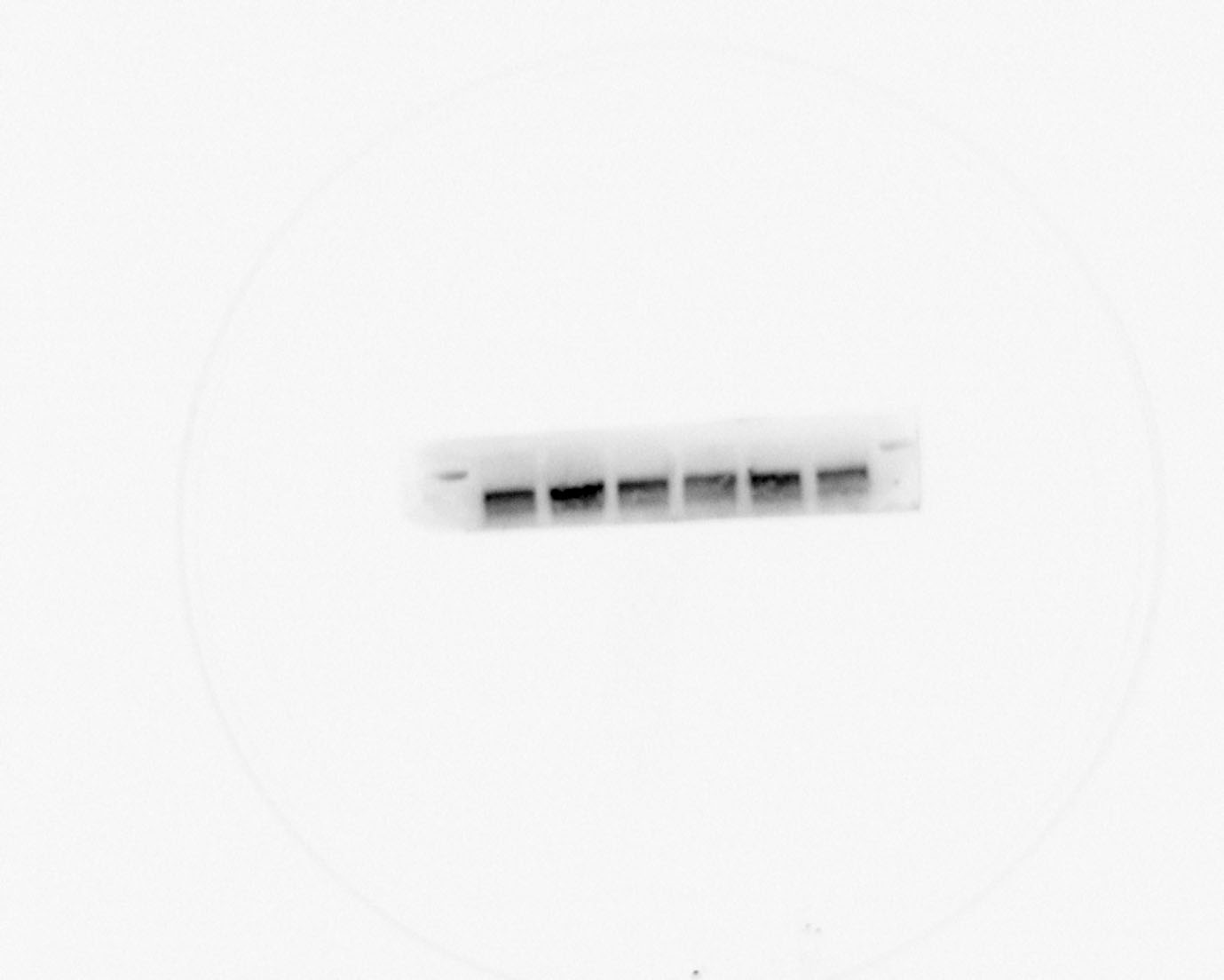

Supplement: Supplementary file 8 [file Image7.JPEG]

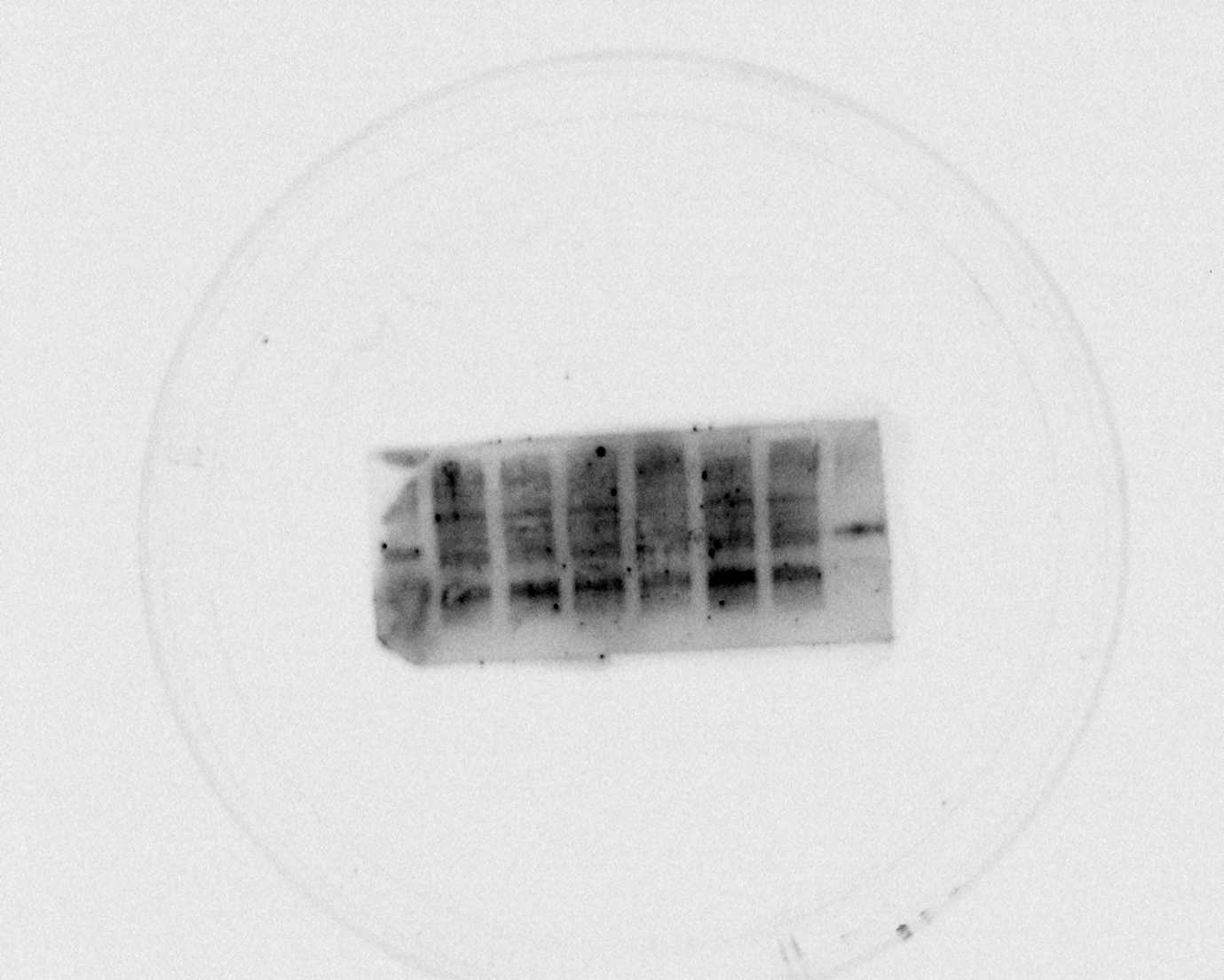

Supplement: Supplementary file 9 [file Image13.TIF]

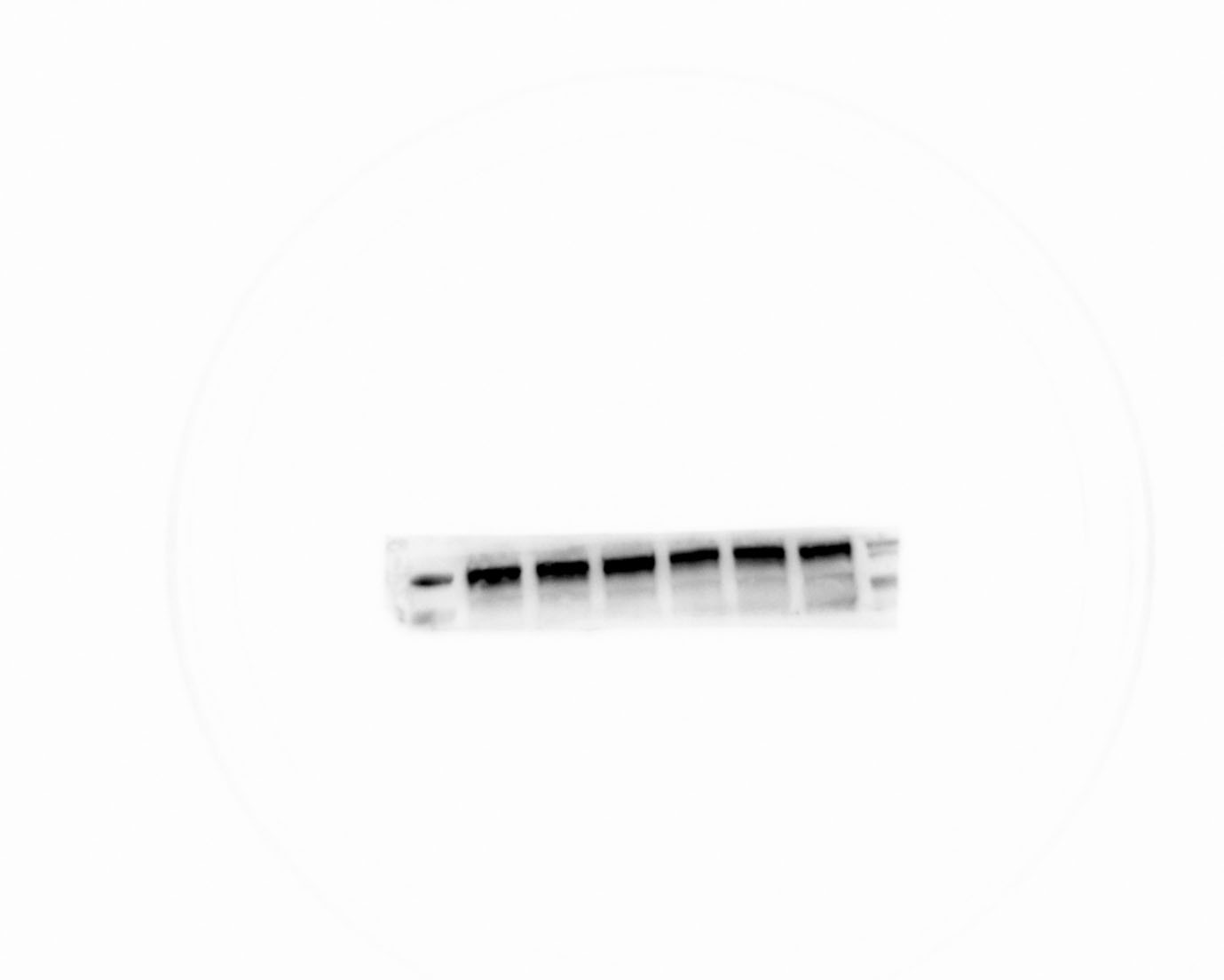

Supplement: Supplementary file 10 [file Image10.JPEG]

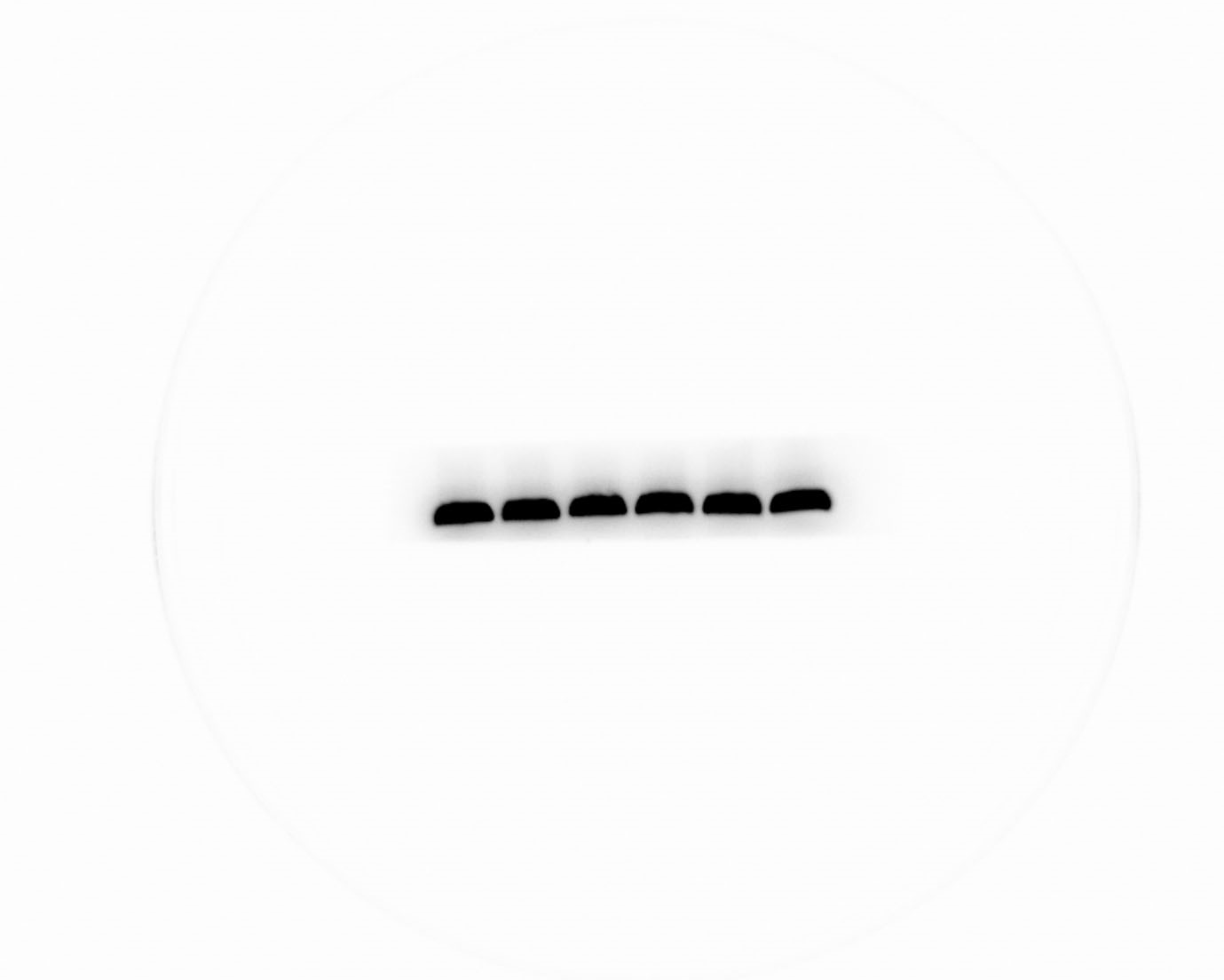

Supplement: Supplementary file 11 [file Image1.TIF]

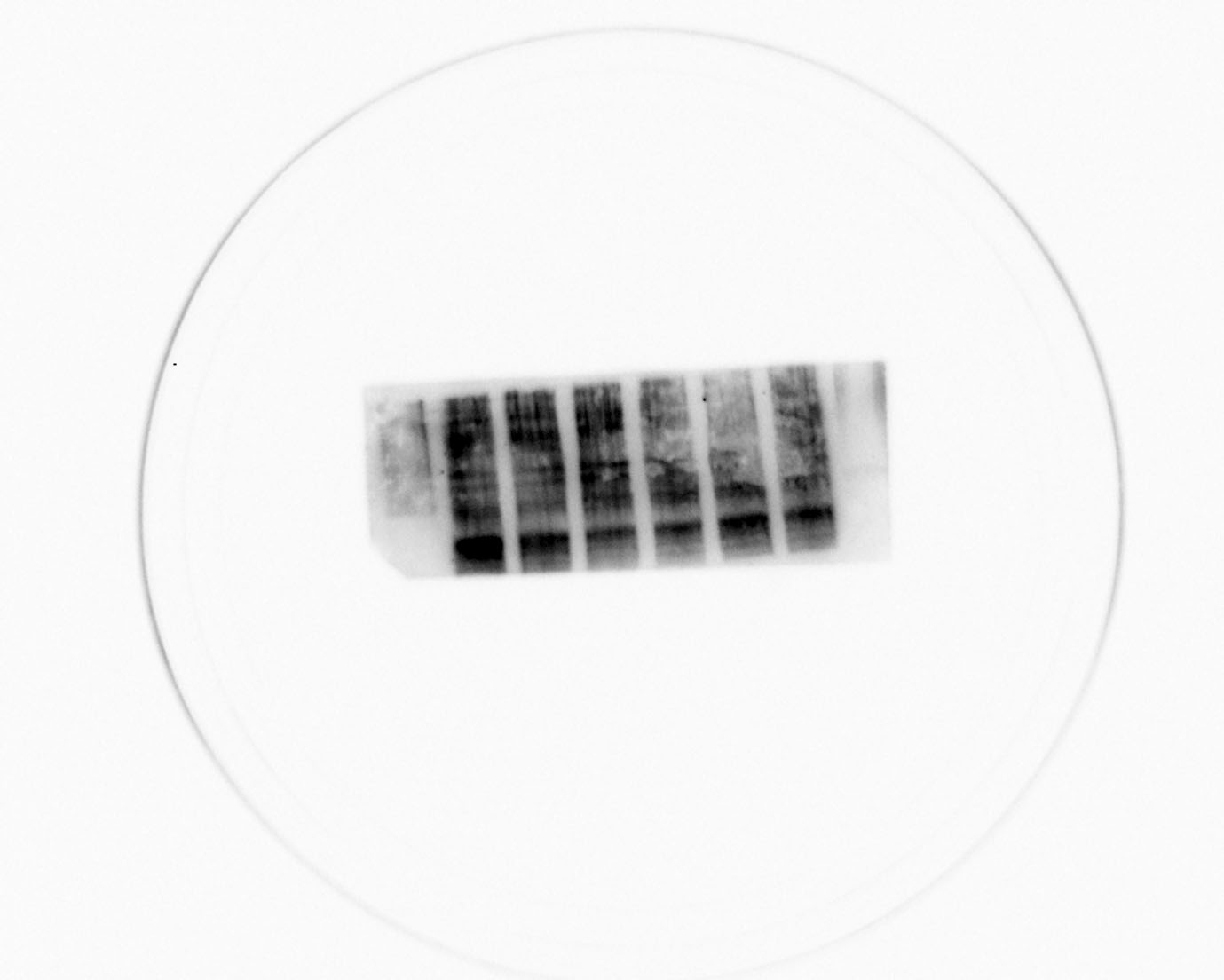

Supplement: Supplementary file 12 [file Image12.JPEG]

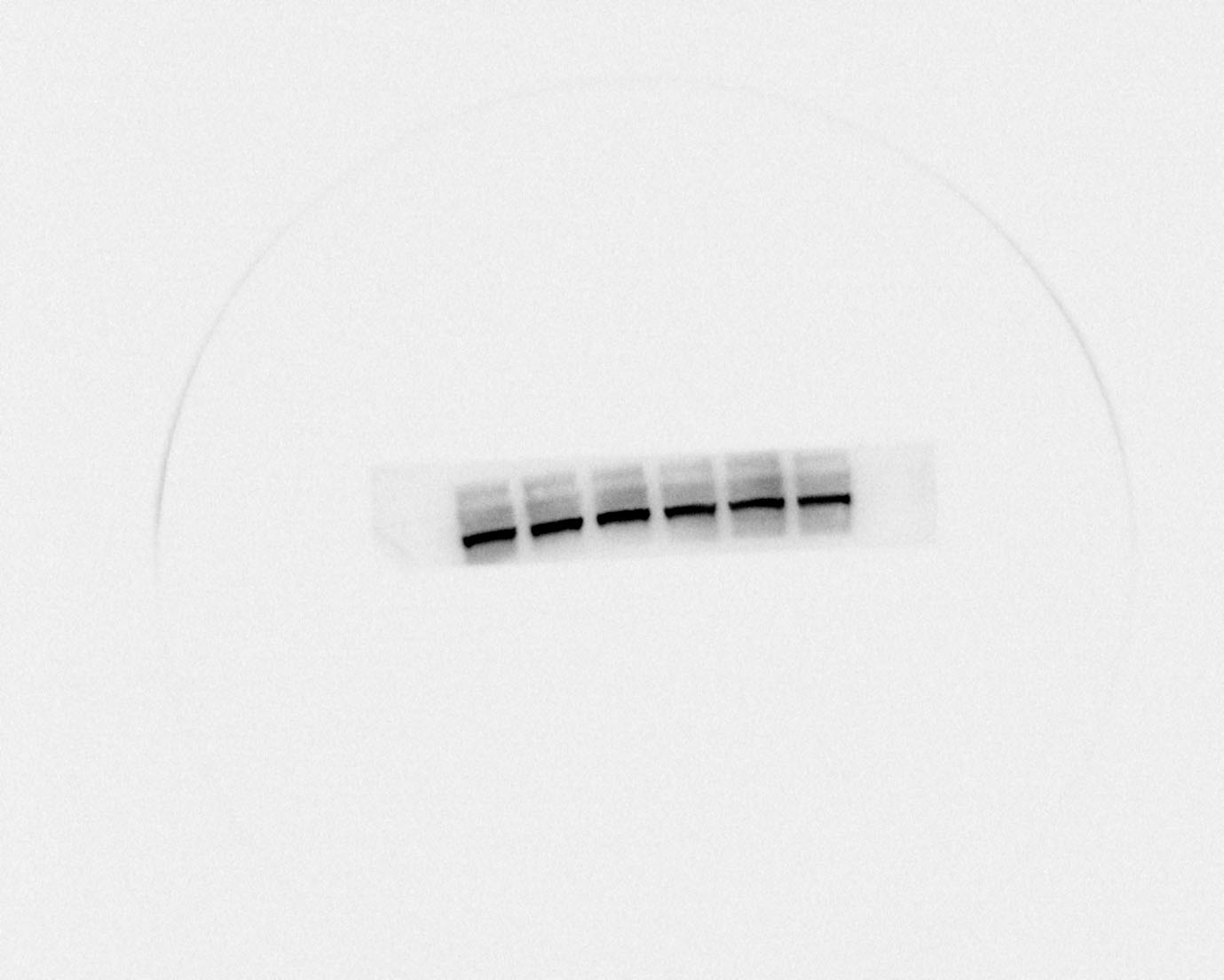

Supplement: Supplementary file 13 [file Image11.JPEG]

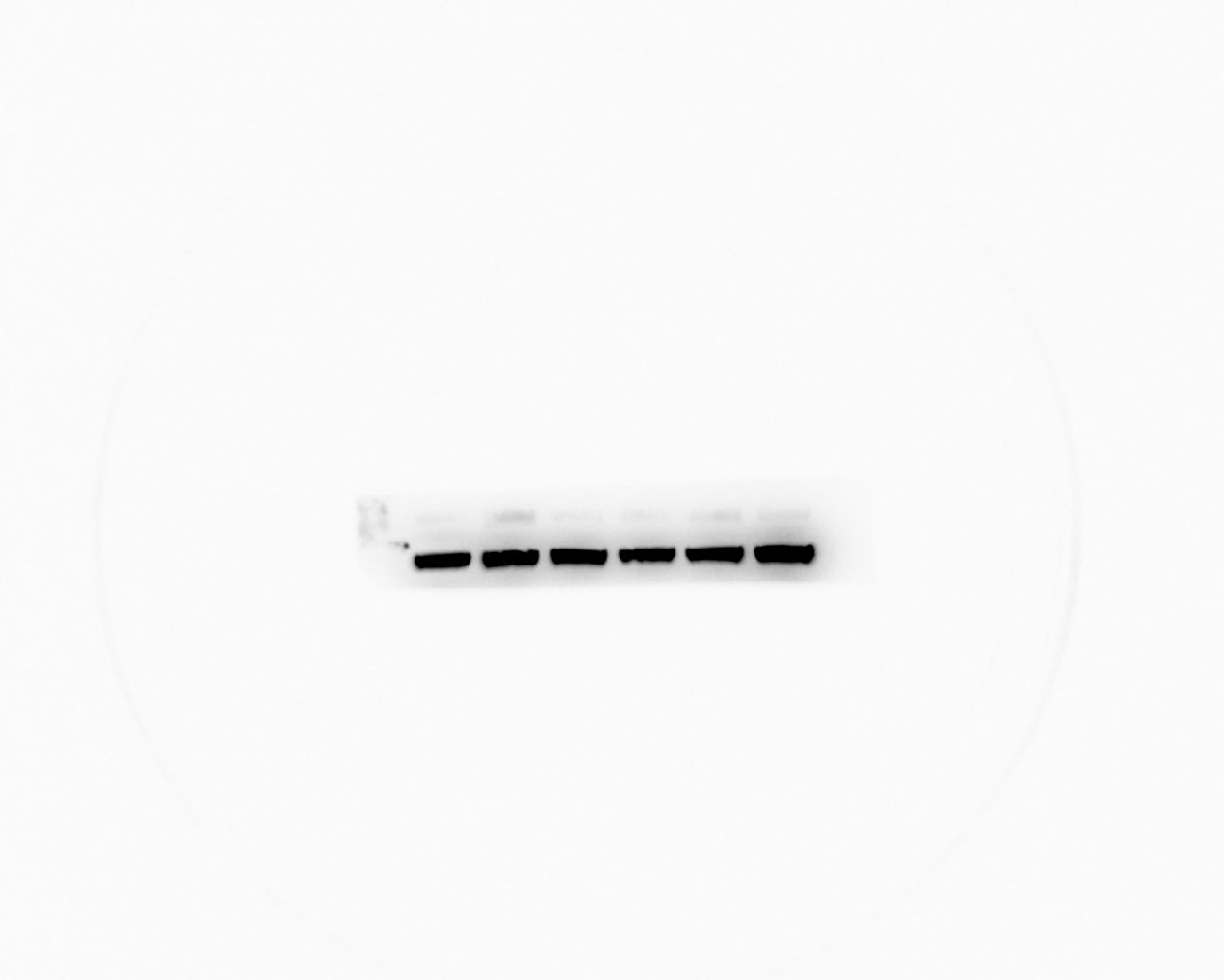

Supplement: Supplementary file 14 [file Image8.JPEG]

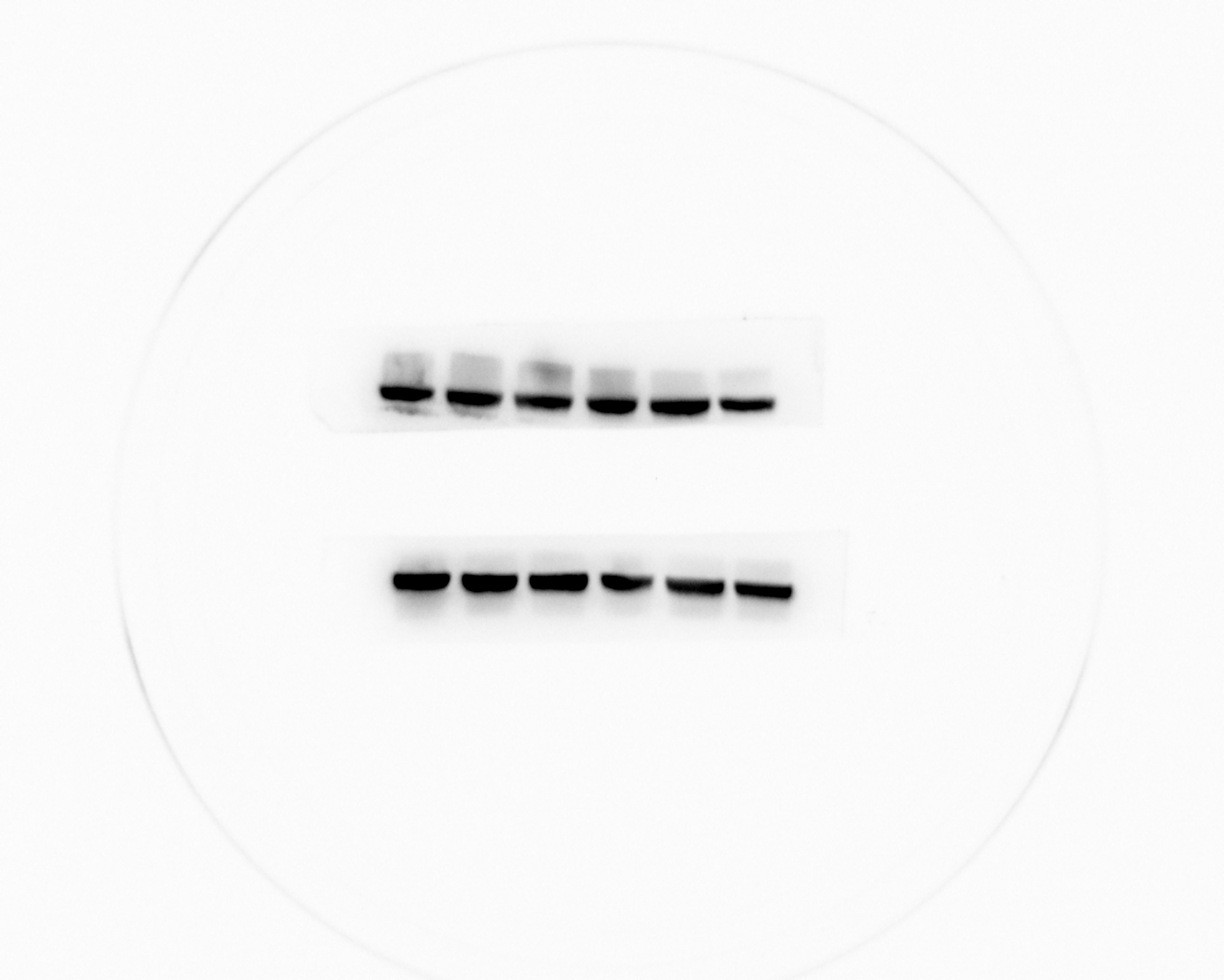

Supplement: Supplementary file 15 [file Image5.TIF]
